# Influence of Genetic West African Ancestry on Metabolomics among Hypertensive Patients

**DOI:** 10.3390/metabo12090783

**Published:** 2022-08-24

**Authors:** Mai Mehanna, Caitrin W. McDonough, Steven M. Smith, Yan Gong, John G. Gums, Arlene B. Chapman, Julie A. Johnson, Rhonda M. Cooper-DeHoff

**Affiliations:** 1 Department of Pharmacotherapy and Translational Research and Center for Pharmacogenomics and Precision Medicine, College of Pharmacy, University of Florida, Gainesville, FL 32610, USA; 2 Department of Pharmaceutical Outcomes & Policy, College of Pharmacy, University of Florida, Gainesville, FL 32610, USA; 3 Department of Medicine, University of Chicago, Chicago, IL 60637, USA

**Keywords:** African ancestry, metabolomics, hypertension, blood pressure

## Abstract

Patients with higher genetic West African ancestry (GWAA) have hypertension (HTN) that is more difficult to treat and have higher rates of cardiovascular diseases (CVD) and differential responses to antihypertensive drugs than those with lower GWAA. The mechanisms underlying these disparities are poorly understood. Using data from 84 ancestry-informative markers in US participants from the Pharmacogenomic Evaluation of Antihypertensive Responses (PEAR) and PEAR-2 trials, the GWAA proportion was estimated. Using multivariable linear regression, the baseline levels of 886 metabolites were compared between PEAR participants with GWAA < 45% and those with GWAA ≥ 45% to identify differential metabolites and metabolic clusters. Metabolites with a false discovery rate (FDR) < 0.2 were used to create metabolic clusters, and a cluster analysis was conducted. Differential clusters were then tested for replication in PEAR-2 participants. We identified 353 differential metabolites (FDR < 0.2) between PEAR participants with GWAA < 45% (*n* = 383) and those with GWAA ≥ 45% (*n* = 250), which were used to create 24 metabolic clusters. Of those, 13 were significantly different between groups (Bonferroni *p* < 0.002). Four clusters, plasmalogen and lysoplasmalogen, sphingolipid metabolism and ceramide, cofactors and vitamins, and the urea cycle, were replicated in PEAR-2 (Bonferroni *p* < 0.0038) and have been previously linked to HTN and CVD. Our findings may give insights into the mechanisms underlying HTN racial disparities.

## 1. Introduction

Overall, Black individuals with hypertension (HTN) have a disease that is more severe and resistant to antihypertensive treatment, poorer blood pressure (BP) control, higher rates of target organ damage and higher mortality rates compared to White individuals with HTN [1,2]. Evidence also suggests that Black patients achieve a better BP response to diuretics and calcium channel blockers, whereas White patients respond better to β-blockers, angiotensin-converting enzyme (ACE) inhibitors and angiotensin II receptor blockers (ARBs) [3]. The mechanisms underlying these racial disparities in HTN and BP response remain poorly understood.

Hypertension is a complex disease with several heterogeneous pathophysiological pathways. These pathways are believed to be regulated by complex interactions between genetic and environmental factors [4,5,6]. Over 1000 genetic loci associated with BP have been previously identified from numerous genome-wide association studies, with some linked to environmental factors such as obesity, sedentary hours, diet, smoking status and alcohol intake [5,6,7,8]. Metabolomics, the study of the intermediates or products of metabolism within cells, is an emerging promising tool that can reflect both genetic and environmental factors, thus enriching our understanding of the dynamic complex metabolic pathways underlying a disease [9]. Over the past few years, numerous studies have shown that various metabolites and metabolic pathways, including fatty acids, phospholipids and metabolites involved in leucine, isoleucine and valine metabolism; tryptophan metabolism; glycine, serine and threonine metabolism; and the urea cycle, are altered in patients with HTN compared to healthy controls [10,11,12,13,14,15,16,17,18]. Additionally, several studies have shed light on metabolic changes that occur upon treatment with antihypertensive agents [19,20,21,22,23,24]. Among these metabolic changes, treatment with thiazide diuretics, β-blockers and calcium channel blockers was associated with increased uric acid and urea cycle metabolites, decreased levels of medium- and long-chain fatty acids, and decreased levels of acyl-carnitines and hexadecanedioate, respectively [19,20,21,23]. Collectively, these studies provide insights on the diverse pathways underlying HTN but have not significantly improved our understanding of HTN racial disparities.

Only a few studies with relatively small sample sizes have explored metabolomic differences between healthy Black and White individuals or between Black and White patients with HTN [25,26,27]. However, these studies reported self- or investigator-defined race, which is a social rather than biological construct and is confounded by racism, socioeconomic inequality and environmental disparities [28,29]. Because US Black and White individuals represent a mixture of West African and European geographic ancestries with diverse frequencies, the biological mechanisms that contribute to racial disparities in HTN may be better informed by using genetically defined ancestry [29,30]. In this study, we aimed to identify the differential metabolites and metabolic clusters between US hypertensive participants with genetic West African ancestry (GWAA) < 45% and those with GWAA ≥ 45%, followed by replication in an independent cohort. We also sought to further test the association between the metabolites within the replicated clusters and the proportion of GWAA among hypertensive patients with GWAA ≥ 45% to assess whether the metabolomic differences are driven by ancestral biological disparities or by other extrinsic and social factors that are different between the two ancestry groups.

## 2. Results

### 2.1. Study Population

The analysis in the current study included baseline data from participants enrolled in the Pharmacogenomic Evaluation of Antihypertensive Responses (PEAR) trial (discovery cohort) and in the PEAR-2 trial (replication cohort). For the 125 participants enrolled in both studies, only data from PEAR-2 were included (Figure S1a). In addition, for PEAR-2 participants who had baseline metabolomics data at two different time points (pre-metoprolol (first time point) and pre-chlorthalidone (second time point)), only the pre-metoprolol data were included in the replication analysis. A total of 633 participants from PEAR and a total of 411 participants from PEAR-2 were included (Figure S1b). The baseline characteristics of the participants from both cohorts are presented in Table 1. Overall, the participants were about 50 years old, on average; a majority were overweight or obese with a mean body mass index of about 31 kg/m^2^, and 50% were women. In both trials, participants with GWAA ≥ 45% had lower baseline plasma renin activity (PRA) compared to those with GWAA < 45%, consistent with the prior literature [3,31,32]. Among PEAR and PEAR-2 participants with lower GWAA, the median GWAA proportion was 1.6% (interquartile range (IQR), 1–3.5%). Of these participants, 2.6% self-identified as Asian or another race. In contrast, among PEAR and PEAR-2 participants with higher GWAA, the median GWAA proportion was 85.1% (IQR, 79–90.6%). Of these participants, 2.3% self-identified as White or other (Figure S2a–d).

### 2.2. Data Processing and Quality Control of PEAR Metabolomics Data

Of the 1223 metabolites (971 known and 252 unknown) detected in the PEAR plasma samples at baseline, 337 metabolites were excluded, including xenobiotics (*n* = 191) and metabolites with >60% missing data (*n* = 146). The remaining 886 metabolites (699 known and 187 unknown) were included in the quality control (QC) steps and final analyses. Principal Component Analysis (PCA) and Standard Euclidean Distance (SED), performed for QC, indicated that 25 participants were considered outliers based on their metabolomics data (Figure S3). The Bland–Altman (BA) method flagged 24 metabolites, each having >5% of the values as outliers (Table S1). Lastly, the top 10% of metabolites with the largest Coefficient of Variation (CV) values (metabolites with the highest variability among PEAR participants) (*n* = 36) were flagged (Table S2). More details on the data processing and QC work are illustrated in Supplementary Material.

### 2.3. Untargeted Metabolomics Analysis

**Figure 1:**
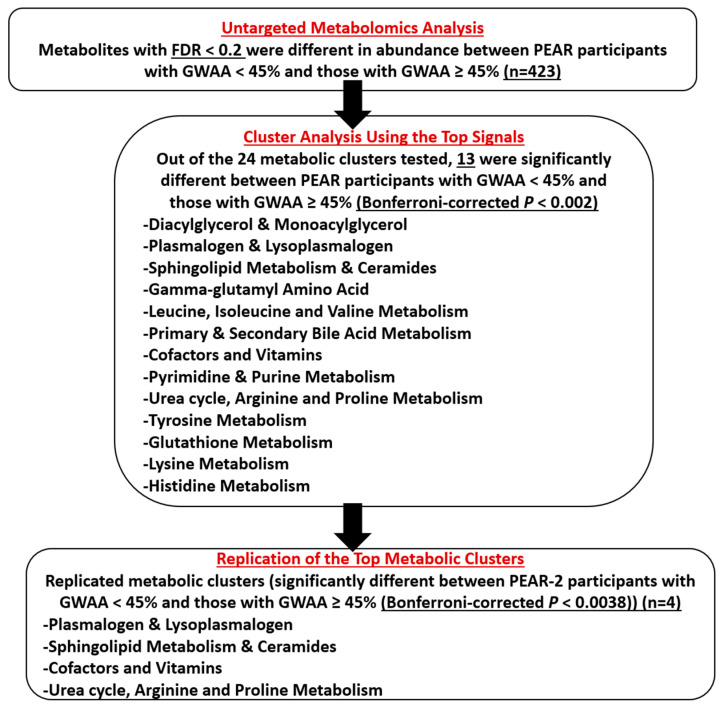
Chart showing the flow of the study results. Abbreviations: FDR, false discovery rate; PEAR, Pharmacogenomic Evaluation of Antihypertensive Responses; GWAA, African ancestry.

Partial least-squares discriminant analysis (PLS-DA) using the baseline log-transformed levels of the 886 metabolites showed a separation between PEAR participants with GWAA < 45% and those with GWAA ≥ 45% (Figure S4). The overall study results are summarized in Figure 1. The baseline log-transformed levels of each of the 886 metabolites were compared between PEAR participants with lower GWAA and those with higher GWAA. Based on a false discovery rate (FDR) < 0.2, a total of 423 metabolites were significantly different between the two ancestry groups (Table S3). Of those, 209 metabolites were more abundant in participants with higher vs. lower GWAA, mainly including amino acids involved in tyrosine metabolism, as well as plasmalogens and lipids involved in bile acid metabolism. Conversely, 214 metabolites were more abundant in participants with lower vs. higher GWAA, mainly including amino acids involved in glutathione metabolism, lysine metabolism and methionine, cysteine, S-Adenosylmethionine (SAM) and taurine metabolism, as well as diacylglycerols and acyl carnitines. Similar results were obtained after conducting a sensitivity analysis, excluding PEAR participants flagged by PCA (*n* = 2), SED (*n* = 21) and both PCA and SED (*n* = 2) QC steps.

### 2.4. Cluster Analysis Using the Top Signals

Because many of the 423 metabolites identified in the screening phase were highly correlated, we grouped metabolites into metabolic clusters using the Modulated Modularity Clustering (MMC) approach and based on the biological pathways to which these metabolites were mapped. Of the 423 metabolites, a total of 70 (unknown metabolites (*n* = 45), known unclassified metabolites (*n* = 20), an amino acid involved in guanidino and acetamido metabolism (*n* = 1), amino acids involved in polyamine metabolism (*n* = 2) and energy metabolites (*n* = 2)) were excluded since they did not cluster well with any of the known classified metabolites. The remaining 353 metabolites were used to create a total of 24 metabolic clusters (Table S4).

**Figure 2:**
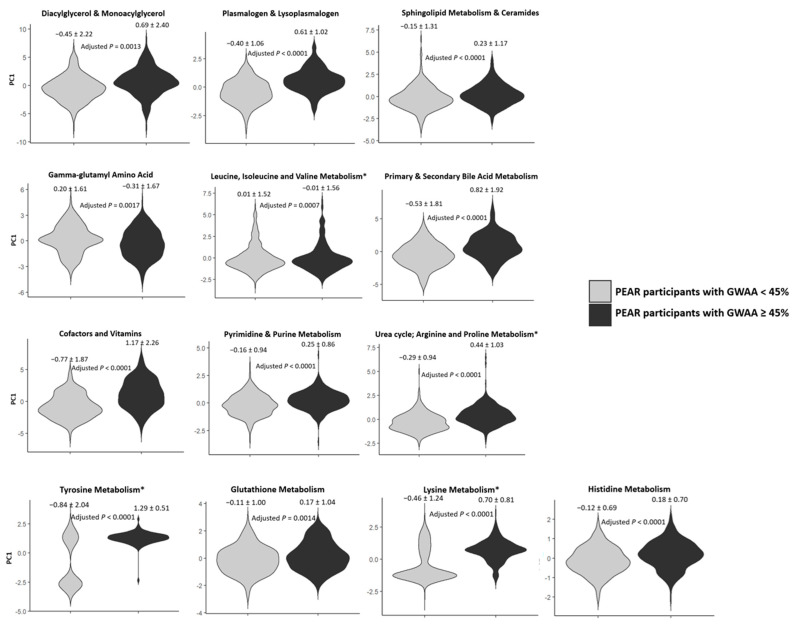
Violin plots comparing PC1 values of each of the 13 significant metabolic clusters between PEAR participants with GWAA < 45% and those with GWAA ≥ 45%. Gray bars represent PEAR participants with GWAA < 45%, whereas the black bars represent PEAR participants with GWAA ≥ 45%. The adjusted *p*-values (Bonferroni-corrected) were calculated using multivariable linear regression models adjusted for age, sex, recruitment site, baseline log-transformed PRA and batch effect. * Metabolic clusters with highly skewed or bimodal PC1 distribution (*n* = 4) (flagged): leucine, isoleucine and valine metabolism; urea cycle/arginine and proline metabolism; tyrosine metabolism and lysine metabolism. Abbreviations: PC, principal component; PEAR, Pharmacogenomic Evaluation of Antihypertensive Responses; GWAA, Genetic West African ancestry.

Cluster analysis using multivariable linear regression showed that 13 of the 24 metabolic clusters achieved Bonferroni-corrected *p* < 0.002. The principal component 1 (PC1) values for each of these 13 metabolic clusters were significantly different between PEAR participants with lower GWAA and those with higher GWAA. Although all 13 clusters were moved forward to the replication phase, the PC1 distribution of four clusters was either highly skewed or bimodal and was flagged. The remaining nine significant non-flagged clusters were diacylglycerol and monoacylglycerol, plasmalogen and lysoplasmalogen, sphingolipid metabolism and ceramides, gamma-glutamyl amino acid, primary and secondary bile acid metabolism, cofactors and vitamins, pyrimidine and purine metabolism, glutathione metabolism and histidine metabolism (Table 2 and Figure 2).

### 2.5. Replication of the Top Metabolic Clusters

**Figure 3:**
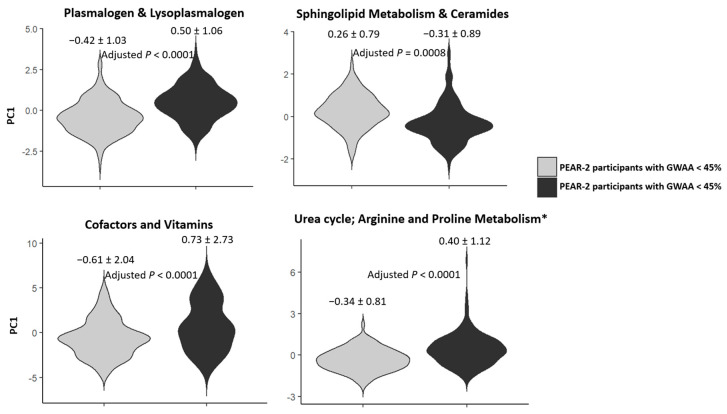
Violin plots comparing PC1 values of each of the 4 replicated metabolic clusters between PEAR-2 participants with GWAA < 45% and those with GWAA ≥ 45%. Gray bars represent PEAR participants with GWAA < 45%, whereas black bars represent PEAR participants with GWAA ≥ 45%. The adjusted *p*-values (Bonferroni-corrected) were calculated using multivariable linear regression models adjusted for age, sex, recruitment site, baseline log-transformed PRA and batch effect. * The metabolic cluster (urea cycle; arginine and proline metabolism) with highly skewed PC1 distribution was flagged. Abbreviations: PC, principal component; PEAR, Pharmacogenomic Evaluation of Antihypertensive Responses; GWAA, Genetic West African ancestry.

Of the 13 metabolic clusters tested for replication in PEAR-2, 4 were successfully replicated. These clusters were plasmalogen and lysoplasmalogen, sphingolipid metabolism and ceramides, cofactors and vitamins and urea cycle–arginine–proline metabolism. The cluster analysis showed that the PC1 values for each of these four metabolic clusters were significantly different between PEAR-2 participants with lower GWAA and those with higher GWAA (Bonferroni-corrected *p* < 0.0038) (Table 3 and Figure 3). In addition, at least 90% of the metabolites included in each of these four replicated clusters had the same direction of effect as in the PEAR study (Table S5).

Only the urea cycle–arginine–proline metabolic cluster was flagged due to its highly skewed PC1 distribution. However, a sensitivity analysis using the Mann–Whitney U test showed that the PC1 values for this cluster were also significantly different between PEAR-2 participants with lower GWAA and those with higher GWAA (*p* < 0.0001). Within the replicated clusters, a total of six metabolites were flagged by the BA and CV QC steps. The details of these results are presented in Supplementary Material.

Within the plasmalogen and lysoplasmalogen cluster, 14 of 15 metabolites were more abundant in hypertensive participants with higher vs. lower GWAA in both PEAR and PEAR-2 (Table S5). Although not statistically significant, our estimates show that as the proportion of GWAA among PEAR and PEAR-2 participants with higher GWAA increases, the levels of 11 of these metabolites also increase (Table S6).

Within the sphingolipid metabolism and ceramides cluster, 13 of 14 metabolites had the same direction of effect in both PEAR studies: 7 were higher in hypertensive participants with higher GWAA, whereas 6 were higher in those with lower GWAA (Table S5).

Within the cofactors and vitamins cluster, 20 of 22 metabolites had the same direction of effect in both PEAR studies. Participants with lower GWAA had higher levels of the three metabolites involved in tocopherol metabolism and the three metabolites involved in nicotinate and nicotinamide metabolism (Table S5 and Figure S5). In contrast, participants with higher GWAA had higher levels of the three sulfate isomers of piperine (Table S5).

Within the urea cycle–arginine–proline metabolism cluster, all 12 metabolites had the same direction of effect in both PEAR studies. This included arginine, homoarginine, hydroxyproline and homocitrulline, which were all more abundant in participants with higher GWAA than those with lower GWAA (Table S5 and Figure S6).

## 3. Discussion

Racial disparities in HTN are believed to be multifactorial, involving genetic factors, psychosocial stressors, socioeconomic status and other structural or environmental factors [33]. To our knowledge, this is the first study undertaken to explore the metabolomic differences between hypertensive participants with lower and higher GWAA, with findings that might reflect some of these underlying factors. Using data from two independent cohorts, we were able to identify and replicate four metabolic clusters (plasmalogen and lysoplasmalogen, sphingolipid metabolism and ceramides, cofactors and vitamins and urea cycle–arginine–proline metabolism) that differed based on GWAA. We found distinct cluster profiles by comparing hypertensive participants with lower vs. higher GWAA, as well as differences in the abundance of metabolites within each cluster.

Our data showed that the plasmalogen and lysoplasmalogen cluster was significantly different between hypertensive participants with lower and those with higher GWAA in both PEAR and PEAR-2. Within that cluster, 14 of 15 metabolites were present at higher levels in participants with higher GWAA compared to those with lower GWAA. Plasmalogens are a class of membrane glycerophospholipids, which represent up to 20% of total phospholipids in humans. Lysoplasmalogens are produced via degradation of plasmalogens by the action of phospholipase A2 [34]. In a prior study investigating metabolite differences after a dietary intervention, several of these lipids, including 1-(1-enyl-palmitoyl)-2-arachidonoyl-GPC (P-16:0/20:4)*, 1-(1-enyl-palmitoyl)-2-linoleoyl-GPE (P-16:0/18:2)* and 1-(1-enyl-stearoyl)-2-linoleoyl-GPE (P-18:0/18:2)* (all were more abundant in participants with higher vs. lower GWAA in our study), were found to be lower among participants randomly assigned to the Dietary Approaches to Stop Hypertension (DASH) diet compared to those randomly assigned to the control diet [35]. In addition, Spears et al. showed that decreased circulating plasmalogens in mice were associated with decreased BP [36]. Additionally, in a population-based Chinese cohort, two plasmalogens within this cluster, 1-(1-enyl-oleoyl)-GPE (P-18:1)* and 1-(1-enylpalmitoyl)-GPE (P-16:0), were shown to be involved in BP regulation [37]. These studies suggest a potential positive relationship between these metabolites and BP. We also found that most of the metabolites within the plasmalogen and lysoplasmalogen cluster were positively associated with the proportion of GWAA, which might indicate potential biological differences that complement other differential environmental or social factors between the two ancestry groups.

We demonstrated that the sphingolipid metabolism and ceramides cluster was significantly distinct between hypertensive participants with lower GWAA and those with higher GWAA in both PEAR studies. Among healthy subjects, Hammad et al. showed that African Americans had higher levels of most sphingomyelins and ceramides tested compared to White Americans [38]. Sphingolipids and ceramides were found to be involved in the regulation of vascular contractility via regulation of nitric oxide (NO) and have also been positively linked to HTN, resistant HTN and BP in several previous studies [24,37,39,40,41]. In addition, among the metabolites within our replicated sphingolipid metabolism and ceramides cluster, sphingomyelin (d18:2/24:2)*, sphingomyelin (d18:1/21:0, d17:1/22:0, d16:1/23:0)*, sphingomyelin (d18:2/14:0, d18:1/14:1)*, sphingomyelin (d18:2/16:0, d18:1/16:1)* and sphingomyelin (d18:1/14:0, d16:1/16:0)* have been previously significantly positively associated with BP [37]. Additionally, we and others previously demonstrated that N24:2 sphingomyelin, sphingosine 1-phosphate and sphingomyelin C24:1 were associated with BP response to the thiazide diuretic hydrochlorothiazide (HCTZ) [21,42,43]. These studies reflect the relevance of these lipids to HTN pathophysiology and HCTZ BP response.

Moreover, our data indicate that the cofactors and vitamins cluster was significantly different between hypertensive participants with lower GWAA and those with higher GWAA in both PEAR cohorts. Within that cluster, three of four metabolites tested within the tocopherol (vitamin E) metabolic pathway (including alpha-tocopherol) and all three metabolites tested within the nicotinate and nicotinamide (vitamin B3) metabolic pathway were higher in participants with lower GWAA than in those with higher GWAA. Consistently, data from the National Health and Nutrition Examination Survey (NHANES) (1999–2000) showed that White Americans had significantly higher levels of serum alpha-tocopherol compared to African Americans [44]. Previous animal and human studies also showed that the metabolites within these two pathways were inversely correlated with HTN, BP and cardiovascular disease (CVD)-related mortality [16,45,46,47,48,49]. Alpha-tocopherol has been previously shown to be associated with a decreased risk of incident HTN as well as overall mortality [16,46]. In addition, nicotinamide resulted in a decrease in BP in endothelial nitric oxide synthase (eNOS)-null mice, which might indicate its potential beneficial effect in hypertensive patients with impaired eNOS [48]. Further studies are warranted to assess whether these metabolites contribute to racial disparities in HTN severity and outcomes, as well as in antihypertensive response [1]. Interestingly, our data suggest that alpha-tocopherol levels were also negatively associated with the proportion of GWAA, which might reflect biological differences between the two ancestry groups. In line with this finding, several genetic variants (located within genes involved in vitamin E uptake and catabolism) that have been associated with lower alpha-tocopherol levels have higher frequencies in African Americans than in Europeans [50,51,52].

We also replicated the urea cycle–arginine–proline metabolism cluster. Participants with higher GWAA had a higher abundance of arginine and homoarginine compared to those with lower GWAA. Previous studies yielded inconsistent results on the levels of these metabolites in Black vs. White participants [53,54,55]. Both arginine and homoarginine increase the bioavailability of the vasorelaxant NO. However, the literature shows controversial findings on the beneficial effects of arginine on BP and other CVD risk factors [10,14,54,56,57,58,59]. Additionally, homoarginine was previously associated with an increased risk of HTN, dyslipidemia, insulin resistance and elevated BP [54,60]. Due to the controversy of the relationship of arginine and homoarginine with HTN, BP and CVD, the role of these metabolites in HTN pathophysiology remains unclear, and future studies are warranted.

Within the replicated urea cycle cluster, we found that participants with higher GWAA had a higher abundance of hydroxyproline and homocitrulline compared to those with lower GWAA. Consistent with our findings, Mels et al. showed that Black participants had higher hydroxyproline levels compared with White participants [26]. Another study demonstrated that methylation of the upstream gene to hydroxyproline (prolyl 4-hydroxylase subunit alpha 3 or *P4HA3*) was associated with systolic and diastolic BP in South Asians [61]. Moreover, studies demonstrated that hydroxyproline was linked to heart failure in patients with HTN, and that homocitrulline was associated with an increased risk of CVD, renal damage and death [62,63,64,65,66]. We previously showed that increased hydroxyproline levels upon treatment with the β-blocker atenolol were associated with poorer BP response in Black individuals but not White individuals with HTN [23,67]. Future studies are necessary to assess whether these metabolites contribute to racial disparities in HTN severity and outcomes, as well as in BP response.

Our study has several strengths. First, the results were generated using data from two independent cohorts of highly phenotyped patients with HTN who did not have other chronic diseases, thus reducing potential confounding effects on our findings. We also used genetically defined ancestry, rather than using self-identified race, which may be a more accurate reflection of ancestry. Our study also has several limitations. First is the unavailability of several PEAR metabolic signals in the PEAR-2 dataset. This might have resulted in missing important metabolomic differences between the two ancestry groups. Second, the sample size of PEAR and PEAR-2 participants with higher GWAA was relatively small, which might have limited our power to identify the biological relevance of the metabolites identified within replicated clusters. Third, we identified several unnamed metabolites within our replicated clusters that have unknown implications, and thus, we are not currently able to speculate on insights pertaining to these metabolites.

## 4. Materials and Methods

### 4.1. Study Design and Participants

The primary analysis in the current study included baseline data from participants recruited as part of the PEAR clinical trial (discovery cohort) and as part of the PEAR-2 trial (replication cohort). Both PEAR studies were prospective, multicenter clinical trials conducted in accordance with the Declaration of Helsinki. The protocols were approved by the institutional review boards at all participating US sites (University of Florida in Gainesville, FL; Mayo Clinic in Rochester, MN; and Emory University in Atlanta, GA). All participants provided voluntary written informed consent prior to participation. Further details about study designs are described below and in Supplementary Material.

PEAR and PEAR-2 have been previously described in detail [68,69]. Briefly, adult participants of any race and ethnicity with uncomplicated mild-to-moderate primary HTN were recruited. Participants with secondary HTN, isolated systolic HTN, CVD, diabetes mellitus, or renal or hepatic dysfunction were excluded. Pregnant and lactating women were also excluded. For participants receiving HTN treatment at the time of enrollment, all antihypertensive drugs were discontinued prior to treatment with the protocol-specified antihypertensive drugs (β-blockers and thiazide diuretics), with a washout period of 4–6 weeks. In both trials, pre- and post-treatment BPs were measured, and blood samples were collected at baseline to conduct genotyping and global metabolomics profiling.

PEAR was a randomized, open-label clinical trial (clinicaltrials.gov identifier: NCT00246519), where participants were randomly assigned to either atenolol (β-blocker) followed by HCTZ (thiazide diuretic) as an add-on therapy or HCTZ followed by atenolol as an add-on therapy. PEAR-2 was an open-label, sequential clinical trial (clinicaltrials.gov identifier: NCT01203852), where participants were sequentially treated with metoprolol (a β-blocker) monotherapy, followed by chlorthalidone (a thiazide-like diuretic) monotherapy, with a 4-week washout in between.

### 4.2. Genotyping and Estimation of GWAA

In PEAR, DNA samples were genotyped for ≈1 million single nucleotide polymorphisms (SNPs) using the Illumina Human Omni 1MQuad BeadChip (Illumina, San Diego, CA, USA), and DNA samples from PEAR-2 participants were genotyped for ≈2.5 million SNPs using the Illumina Human Omni2.5S BeadChip (Illumina) [70]. The QC procedures conducted on the genetic data from both studies have been previously described [71].

The global proportion of West GWAA for each PEAR and PEAR-2 participant was estimated using genetic ancestry-informative markers (AIMs), which are defined as SNPs that have largely different frequencies between populations. Evidence suggests that using as few as 64 AIMs can accurately estimate individual genetic ancestry [72]. In this study, we used genome-wide directly genotyped data of 84 AIMs, selected from two sets of previously validated AIMs [72,73]. Our AIMs were selected based on at least 30% difference in allele frequency between the African and the European populations and on the availability of their genotype data in PEAR and PEAR-2 (Table S7) [74]. Using the genotype data of 90 Yoruba individuals (representing GWAA) and 90 European American individuals from the HapMap database, who served as the parental reference populations, the global proportion of GWAA was estimated for each PEAR and PEAR-2 participant [30] using STRUCTURE software with the parameter K set to two populations [75]. The software models the probability of the observed genotypes given the individual ancestry proportions and the ancestral population allele frequencies using traditional Bayesian methods [76].

### 4.3. Untargeted Metabolomics Profiling

Baseline fasting plasma samples from PEAR and PEAR-2 participants were used to perform untargeted/global metabolomics profiling by Metabolon, Inc., Durham, NC, USA, using ultrahigh-performance liquid chromatography–tandem mass spectroscopy (UPLC-MS/MS) (Waters, Milford, MA, USA) [77]. This process has been previously described in detail [43]. Briefly, samples were divided into aliquots and stored at −80° Celsius until processed. At the time of the analysis, an aliquot was thawed, extracted and centrifuged to recover the metabolites. The extract was then divided into five aliquots. Two aliquots were analyzed by two separate reverse phases (RP)/UPLC-MS/MS with positive ion mode electrospray ionization (ESI); one was chromatographically optimized for more hydrophilic compounds, and the other was optimized for more hydrophobic compounds. The third aliquot was analyzed by RP/UPLC-MS/MS with negative ion mode ESI. The fourth aliquot was analyzed using hydrophilic interaction liquid chromatography (HILIC)/UPLC-MS/MS with negative ion mode ESI. The fifth aliquot was reserved for backup. The peaks of each metabolite were quantified using the area under the curve. Metabolites were then identified by comparison to library entries of purified authenticated standards or recurrent unknown entries. All molecules present in this library have information on the retention time/index (RI), mass-to-charge ratio (*m*/*z*) and chromatographic data. Each metabolite was corrected in run-day blocks by normalizing each data point proportionately.

### 4.4. Data Processing and QC of PEAR Metabolomics Data

MetaboAnalyst 5.0, an open-source R-based program for metabolomics, and Galaxy Southeast Center for Integrated Metabolomics (SECIM) tools were used to perform data processing and QC [78,79]. This work is presented in detail in Supplementary Material. In brief, all xenobiotics and metabolites with greater than 60% missingness were excluded. Non-imputed data of the remaining metabolites were included in the QC steps and in the main analysis. Imputation was conducted using the K-nearest neighbor (KNN) algorithm only to perform the PCA, which was used to identify clustering or extreme outliers among PEAR participants based on their metabolomics data. Potential outliers were also identified using SED values. The concordance of the metabolomics data between pairs of PEAR participants within each subgroup was assessed using the BA method [80]. In addition, CV was calculated to assess the consistency of metabolites across participants.

### 4.5. Statistical Analyses

Baseline characteristics of the PEAR and PEAR-2 participants are presented using descriptive statistics. Data for continuous variables are summarized as means with standard deviations (SDs), except for baseline PRA and the proportion of GWAA, which were not normally distributed and are instead summarized as median with IQR. Normally distributed continuous variables were compared between participants with GWAA < 45% and those with GWAA ≥ 45% using the independent *t*-test, whereas the non-normally distributed variables were compared using the Mann–Whitney U test. Data for categorical variables were summarized as numbers and percentages and were compared between the two ancestry groups using the Chi-square test. Metabolomics data in the PEAR studies were not normally distributed. Therefore, log-transformed metabolomics data were used in all analyses. In this study, we used a 3-phase analytic approach, as outlined below. All statistical analyses were conducted in SAS (version 9.4; SAS Institute Inc., Cary, NC, USA) and R Statistical Software (Foundation for Statistical Computing, Vienna, Austria).

#### 4.5.1. Untargeted Metabolomics Analysis (Screening Phase)

After data processing, using the baseline log-transformed levels of the remaining metabolites, multivariate analysis was performed using PLS-DA to detect whether there is a separation between PEAR participants with GWAA < 45% and those with GWAA ≥ 45%. We used the 45% GWAA cutoff based on two criteria: (1) based on prior published work that studied the genetic ancestry of over 5000 self-reported US African Americans and found that ~90% of that population had African ancestry of 44–46% [81] and (2) based on our PEAR data, which showed that the highest GWAA proportion among the PCA-identified White participants was about 40%. Then, the baseline log-transformed levels (in terms of raw area counts, non-imputed data) of each metabolite included in the analysis were compared between PEAR participants with GWAA < 45% and those with GWAA ≥ 45%. This comparison was conducted using multivariable linear regression models adjusted for sex, age, recruitment site, baseline log-transformed PRA and batch effect. Missing values for each tested metabolite were ignored, resulting in a different sample size for each metabolite. Metabolites with FDR < 0.2 were identified and moved forward to the next phase. We used a less stringent FDR threshold since this was a screening phase, in which we were more concerned about type II rather than type I error. A sensitivity analysis was also conducted, excluding PEAR participants flagged by the PCA and SED QC steps. Metabolites flagged by the BA or CV QC steps were included in the analyses but planned to be further investigated if any of them were included within any of the replicated metabolic clusters.

#### 4.5.2. Cluster Analysis Using the Top Signal(s) (Discovery Phase)

To reduce the number of correlated metabolites identified in the screening phase, we used two different approaches to cluster these metabolites into metabolic clusters. The first approach was based on the MMC, which was used to assess pairwise Pearson correlations, mainly between the unknown and known classified metabolites [82]. The second approach was based on biology (the biochemical pathways to which the metabolites are mapped). For example, all of the metabolites involved in the same metabolic pathway based on the biochemical pathway information provided by Metabolon, Inc. (sphingolipid metabolism, tyrosine metabolism, etc.) were grouped within the same metabolic cluster. Additionally, we grouped biologically related metabolites/pathways within the same clusters (grouping diacylglycerols with monoacylglycerols, grouping ceramides with metabolites involved in sphingolipid metabolism, etc.). A cluster analysis was then performed on each of the created metabolic clusters. This process entailed first conducting PCA on each of these clusters by using the KNN-imputed baseline log-transformed metabolomics data and then by comparing the PC1 values between PEAR participants with lower GWAA and those with higher GWAA. This comparison was made using multivariable linear regression, where PC1 for each metabolic cluster was the dependent variable, and the categorical GWAA was the independent variable of interest, while adjusting for the covariates sex, age, recruitment site, baseline log-transformed PRA and batch effect. The normality and skewness of the PC1 values for each cluster were checked by using histograms and the Shapiro–Wilk test and by assessing the degree of skewness. Linear regression models are relatively robust to the violation of the normality assumption, except if the distribution is highly skewed [83]. Therefore, only metabolic clusters with a highly skewed PC1 distribution (determined by a degree of skewness of > +1 or <−1) or a bimodal PC1 distribution were flagged. Violin plots were generated using the ggplot2 R package (https://cran.r-project.org/web/packages/ggplot2/index.html) (accessed on 25 July 2022) to represent the comparison of PC1 values between PEAR participants with lower GWAA and those with higher GWAA. Metabolic clusters with a Bonferroni-corrected *p* were identified and moved forward to the replication phase.

#### 4.5.3. Replication Phase of the Top Metabolic Cluster(s)

The metabolic clusters identified in the discovery phase were tested for replication in an independent cohort (PEAR-2 study). Replication was performed using the same cluster analysis described above by conducting PCA on the identified clusters and then comparing the PC1 values for each cluster between PEAR-2 participants with lower GWAA and those with higher GWAA. Data normality and skewness of the PC1 values for each cluster were also checked as previously described. Additionally, the direction of effect of each metabolite within each cluster tested for replication was assessed by comparing baseline log-transformed levels of metabolites between PEAR-2 participants with lower GWAA and those with higher GWAA using multivariable linear regression, adjusting for sex, age, recruitment site, baseline log-transformed PRA and batch effect. Violin plots were also used to represent the comparison of PC1 values between PEAR-2 participants with lower GWAA and those with higher GWAA. Metabolic clusters were considered successfully replicated if they reached the Bonferroni-corrected p and had at least 90% of their metabolites with the same direction of effect as in the PEAR discovery cohort. A sensitivity analysis was also performed on successfully replicated clusters that were flagged due to a highly skewed or bimodal PC1 distribution. This was carried out by comparing the PC1 values between PEAR-2 participants with lower GWAA and those with higher GWAA using the Mann–Whitney U test.

Additionally, for the replicated metabolic clusters, we tested whether these metabolomic differences are mainly driven by ancestral biological disparities or by other extrinsic and social factors that are different between the two ancestry groups. To do so, we tested the association between each metabolite within each replicated cluster and the proportion of GWAA among both PEAR and PEAR-2 participants with higher GWAA using multivariable linear regression, adjusted for sex, age, recruitment site, baseline log-transformed PRA and batch effect. We restricted this analysis to participants with higher GWAA because the proportion of GWAA was only normally distributed in these participants (Figure S2).

## 5. Conclusions

In conclusion, our study sheds light on several metabolites and metabolic pathways that are significantly distinct between US hypertensive patients with lower GWAA and those with higher GWAA. The prior literature along with our novel findings indicates that many of these metabolomic differences might be involved in the mechanisms underlying HTN. Further studies on the identified metabolites and metabolic pathways are needed to confirm our findings and to explore their potential role in the underlying observed racial disparities in HTN severity, outcomes and BP response. A better understanding of these disparities might result in a more effective personalization of antihypertensive treatment for patients with lower vs. higher GWAA. This could help in eliminating the racial disparities currently seen in HTN, leading to better BP control and a reduction in CVD.

## Figures and Tables

**Table 1:** Baseline characteristics of participants included from PEAR and PEAR-2 studies.

	PEAR			PEAR-2		
Variable	Participants with GWAA < 45% (*n* = 383)	Participants with GWAA ≥ 45% (*n* = 250)	*p*-Value	Participants with GWAA < 45% (*n* = 224)	Participants with GWAA ≥ 45% (*n* = 187)	*p*-Value
Age, years	50.0 ± 9.8	47.3 ± 8.9	0.0005	50.8 ± 9.0	50.2 ± 9.1	0.5
Females, N (%)	163 (42.6%)	165 (66%)	<0.0001	98 (43.8%)	100 (53.5%)	0.05
BMI, kg/m^2^	30.5 ± 5.4	31.5 ± 6.0	0.02	30.8 ± 5.0	31.1 ± 5.4	0.5
GWAA (%)	1.6% (1–3.4%)	85.4% (78.8–90.6%)	<0.0001	1.5% (0.9–3.6%)	84.8% (79.2–90.5%)	<0.0001
Baseline PRA, ng/mL/h	0.9 (0.5–1.5)	0.4 (0.2–0.6)	<0.0001	0.9 (0.5–1.8)	0.4 (0.2–0.7)	<0.0001
Baseline SBP, mmHg	151.2 ± 12.2	151.4 ± 12.9	0.9	149.9 ± 12.4	150.6 ± 13.1	0.5
Baseline DBP, mmHg	98.0 ± 5.7	99.1 ± 6.7	0.03	98.0 ± 5.3	98.7 ± 6.0	0.2

All normally distributed continuous variables are summarized as mean ± SD and were compared using the independent *t*-test. Exceptions are the baseline PRA and % GWAA, which are not normally distributed; they are summarized as median with IQR and were compared using the Mann–Whitney U test. Discrete variables are summarized as N (percentage) and were compared using the Chi-square test. Abbreviations: PEAR, Pharmacogenomic Evaluation of Antihypertensive Responses; GWAA, Genetic West African ancestry; BMI, body mass index; PRA, plasma renin activity; SBP, systolic blood pressure; DBP, diastolic blood pressure; SD, standard deviation; IQR, interquartile range.

**Table 2:** Results of the cluster analysis conducted using PEAR discovery cohort on the 24 metabolic clusters.

Metabolic Cluster	Number of PEAR Metabolites Identified from Screening Phase	% Variability Explained by PC1	Regression *p* (Comparing PC1 Values between PEAR Participants with GWAA < 45% and Those with GWAA ≥ 45%)
Diacylglycerol and monoacylglycerol	19	54.7%	0.0013 *
Plasmalogen and lysoplasmalogen	17	44.3%	<0.0001 *
PC, PE, PI, lysophospholipid and phospholipid metabolism	48	27.4% ⁰	0.0034
Sphingolipid metabolism and ceramides	33	30.6%	<0.0001 *
Steroid and sterol	12	34.6%	0.036
Gamma-glutamyl amino acid	9	44.3%	0.0017 *
Leucine, isoleucine and valine metabolism	14	42.5% ⁰	0.0007 *
Primary and secondary bile acid metabolism	24	21.6%	<0.0001 *
Cofactors and vitamins	23	22.6%	<0.0001 *
Pyrimidine and purine metabolism	14	27.4%	<0.0001 *
Urea cycle; arginine and proline metabolism	12	37.1% ⁰	<0.0001 *
Tyrosine metabolism	7	64.8% ⁰	<0.0001 *
Glutamate metabolism	5	43.4%	0.039
Glycine, serine and threonine metabolism	9	40.4%	0.11
Glutathione metabolism	5	59.6%	0.0014 *
Methionine, cysteine, SAM and taurine metabolism	7	37.9%	0.0026
Lysine metabolism	8	47.4% ⁰	<0.0001 *
Alanine and aspartate metabolism	4	50.3%	0.03
Histidine metabolism	4	52.9%	<0.0001 *
Carbohydrate	5	41.9%	0.0043
Creatine metabolism	3	54.0%	0.07
Fatty acid	41	23.9%	0.16
Fatty acid metabolism (acyl carnitine)	21	31.8%	0.0086
Tryptophan metabolism	9	45.3% ⁰	0.36

* This metabolic cluster has a Bonferroni-corrected *p* < 0.002 (0.05/24). ^0^ The PC1 distribution of this metabolic cluster is either highly skewed (degree of skewness is >+1 or <−1) or bimodal (flagged). Abbreviations: PEAR, Pharmacogenomic Evaluation of Antihypertensive Responses; PC1, principal component 1; GWAA, Genetic West African ancestry.

**Table 3:** Results of the cluster analysis conducted using the PEAR-2 replication cohort on the 13 metabolic clusters.

Metabolic Cluster	Number of Metabolites in PEAR-2	% Variability Explained by PC1	Regression *p* (Comparing PC1 Values between PEAR-2 Participants with GWAA < 45% and Those with GWAA ≥ 45%)
Diacylglycerol and monoacylglycerol	5	58.8%	0.05
Plasmalogen and lysoplasmalogen	15	47.2%	<0.0001 *
Sphingolipid metabolism and ceramides	14	42.3%	0.0008 *
Gamma-glutamyl amino acid	7	64.0% ⁰	0.19
Leucine, isoleucine and valine metabolism	12	56.2%	0.57
Primary and secondary bile acid metabolism	22	26.6%	0.05
Cofactors and vitamins	22	28.0%	<0.0001 *
Pyrimidine and purine metabolism	13	27.4%	0.28
Urea cycle; arginine and proline metabolism	12	37.5% ⁰	<0.0001 *
Tyrosine metabolism	5	57.6%	0.009
Glutathione metabolism	4	59.1%	0.6
Lysine metabolism	7	53.8%	0.23
Histidine metabolism	4	46.3%	0.03

* This metabolic cluster has a Bonferroni-corrected *p* < 0.0038 (0.05/13). ⁰ The PC1 distribution of this metabolic cluster is either highly skewed (degree of skewness is > +1 or <−1) or bimodal (flagged). The only replicated flagged cluster was (urea cycle; arginine and proline metabolism), which was also significantly different between PEAR-2 participants with GWAA < 45% and those with GWAA ≥ 45% using the Mann–Whitney U test (*p* < 0.0001). Abbreviations: PEAR, Pharmacogenomic Evaluation of Antihypertensive Responses; PC1, principal component 1; GWAA, Genetic West African ancestry.

## Data Availability

The data presented in this study are available on request from the corresponding author. The data are not publicly available due to ethical and privacy concerns.

## Supplementary Materials

The following supporting information can be downloaded at: https://www.mdpi.com/article/10.3390/metabo12090783/s1. Figure S1: Diagrams showing participants included in this study; Figure S2: Distributions of GWAA proportion among PEAR and PEAR-2 participants with GWAA < 45% and those with GWAA ≥ 45%; Figure S3: PCA scatter plots showing clustering of PEAR participants (*n* = 633) based on the first three PCs; Figure S4: Partial least-squares discriminant analysis (PLS-DA) of the 886 plasma metabolites in PEAR participants with GWAA < 45% (*n* = 383) and PEAR participants with GWAA ≥ 45% (*n* = 250); Table S1: Metabolites flagged by the BA measures and plots in PEAR (*n* = 24); Table S2: The top 10% of metabolites with the largest CV values in PEAR (*n* = 36); Table S3: Classifications of the differential 423 metabolites (FDR < 0.2) between PEAR participants with GWAA < 45% and those with GWAA ≥ 45% (screening phase); Table S4: The 24 metabolic clusters created using the 353 PEAR metabolites identified in the screening phase; Table S5: Metabolites within each of the 4 replicated metabolic clusters; Table S6: Metabolites within replicated clusters having the same direction of effect using the proportion of GWAA among PEAR participants with GWAA ≥ 45% (*n* = 250) and PEAR-2 participants with GWAA ≥ 45% (*n* = 187); Figure S5: The KEGG nicotinate and nicotinamide metabolic pathway; Figure S6: The KEGG arginine and proline metabolic pathway; Table S7: The 84 AIMs used to estimate the proportion of GWAA for each PEAR and PEAR-2 participant.
