# Supplementary material for: Influence of Genetic West African Ancestry on Metabolomics among Hypertensive Patients
Source: Metabolites. 2022 Aug 24;12(9):783. doi: 10.3390/metabo12090783 (PMC9506508; doi:10.3390/metabo12090783)
Supplement: Supplementary file 1 [file metabolites-12-00783-s001.zip › metabolites-1862582-supplementary.pdf]

metabolites

Number of figures: 6
Number of tables: 7

Influence of Genetic West African Ancestry on Metabolomics among Hypertensive Patients

Mai Mehanna [1], Caitrin W. McDonough [1], Steven M. Smith [1,2], Yan Gong [1], John G. Gums [1], Arlene B. Chapman [3], Julie A. Johnson [1] and Rhonda M. Cooper-DeHoff [1,*]

[1] Department of Pharmacotherapy and Translational Research and Center for Pharmacogenomics and Precision Medicine, College of Pharmacy, University of Florida, Gainesville, FL 32610, USA
[2] Department of Pharmaceutical Outcomes & Policy, College of Pharmacy, University of Florida, Gainesville, FL 32610, USA
[3] Department of Medicine, University of Chicago, Chicago, IL 60637, USA
* Correspondence: dehoff@cop.ufl.edu; Tel.: +1-(352)-273-6184

Running Head:

Key Words: African ancestry; metabolomics; hypertension; blood pressure.

Clinical Trial Registry Numbers: NCT01203852; NCT00246519

Methods

Study Design and Participants

The Pharmacogenomics Evaluation of Antihypertensive Responses (PEAR)

We used data from participants recruited as part of PEAR clinical trial in our screening and discovery phases. PEAR was a prospective, multicenter, randomized, open-label clinical trial, conducted at three US sites (University of Florida in Gainesville, FL; Mayo Clinic in Rochester, MN; and Emory University in Atlanta, GA) (clinicaltrials.gov identifier: NCT00246519). Details of this study have been previously described [1]. Participants with uncomplicated mild to moderate essential hypertension (HTN), of any race, aged 17-65 years old were recruited. Exclusion criteria were secondary HTN, systolic blood pressure (SBP)> 180 mmHg or diastolic BP (DBP) > 110 mmHg, isolated systolic HTN, cardiovascular disease, diabetes mellitus, heart rate < 55 beats/ min, renal or hepatic dysfunction. Also, pregnant and lactating women were excluded. After a washout period of about four weeks of any antihypertensive drugs, participants were randomly assigned to either the β-blocker atenolol 50 mg once daily (dose titrated to 100 mg once daily if BP > 120/70 mmHg) or the thiazide diuretic hydrochlorothiazide (HCTZ) 12.5 mg once daily (dose titrated to 25 mg once daily if BP > 120/70 mmHg) for a total of 9 weeks. After monotherapy treatment, if BP remained above the goal, drug from the other treatment arm was added (i.e HCTZ was added for those on atenolol, and vice versa), followed by the same dose titration for another 6-9 weeks of treatment.

PEAR-2

In the current study, we used data from participants recruited as part of PEAR-2 trial in our replication phase. PEAR-2 was a prospective, multicenter, open-label, sequential clinical trial,

conducted at the same US three sites mentioned above (clinicaltrials.gov identifier: NCT01203852). Details of this trial have been previously published [2]. Participants with uncomplicated mild to moderate essential HTN, of any race, aged 18-65 years old were recruited. PEAR-2 exclusion criteria were the same as described above for PEAR. After an average of four-week washout period of any antihypertensive medications, participants were initially treated with the β-blocker metoprolol 50 mg twice daily for two weeks, followed by a dose titration to 100 mg twice daily for an additional six weeks. After a second washout period, participants were then treated with the thiazide-like diuretic chlorthalidone 15 mg once daily, followed by a dose titration to 25 mg once daily for an additional six weeks.

Data Processing and Quality Control on PEAR Metabolomics Data

A total of 1223 metabolites (971 known and 252 unknown) have been detected in PEAR plasma samples at baseline. The known classified metabolites included 351 lipids, 191 xenobiotics, 165 amino acids, 37 nucleotides, 34 peptides, 27 cofactors and vitamins, 26 carbohydrates, 8 energy metabolites and other 132 unclassified metabolites. We performed processing and quality control (QC) on PEAR metabolomics data using MetaboAnalyst 5.0 (an open-source R-based program for metabolomics) and Galaxy Southeast Center for Integrated Metabolomics (SECIM) tools [3,4]. Out of the 1223 metabolites, a total of 337 metabolites were excluded. First, all xenobiotics (n=191) were removed to reduce the environmental confounding effects on our findings. These included drugs' metabolites (n=86), food components (n=42), chemicals (n=21), metabolites involved in benzoate metabolism (n=21), metabolites involved in xanthine metabolism (n=16), tobacco metabolites (n=4) and bacterial/ fungal metabolite (n=1). Additionally, all metabolites with greater than 60% missing

data (n=146) were excluded. Non-imputed data of the remaining 886 metabolites were included in the QC steps and in the main analysis. Imputation using the K-nearest neighbors algorithm was conducted only to perform principal component analysis (PCA) as one of the QC steps. The main objective of QC was to identify and flag outlying participants (based on the metabolomics data) and outlying metabolites. The QC steps are illustrated in detail below.

PCA

The first ten principal components (PCs) explain a total of about 86% of the variability in PEAR metabolomics data. Based on the first three PCs which explain a total of 68%, there was no clustering among PEAR participants. Also, there was no clustering by sex, age group, body mass index (BMI) group, batch, or recruitment site (data not shown). However, four outliers have been identified (flagged) **(Figure S3)**, and a sensitivity analysis (screening phase) was conducted excluding them.

Standard Euclidean Distance (SED)

SED was used to identify the outlying participants based on their metabolic states. SED between each participant and the estimated mean was calculated. A total of 23 participants had the largest SED values and were considered outliers based on their metabolic states. These participants were flagged, and a sensitivity analysis (screening phase) was conducted excluding them.

Bland-Altman (BA)

The BA assesses concordance of the metabolomics data between pairs of participants within specified subgroups [5]. The expectation is that participants within the same subgroup have similar metabolic states. To conduct this QC step, PEAR participants were categorized into the following eight subgroups: male participants aged < 50 years old with BMI < 30 kg/m^2 (n=66), male participants

aged < 50 years old with BMI ≥ 30 kg/m^2 (n=82), male participants aged ≥ 50 years old with BMI < 30 kg/m^2 (n=88), male participants aged ≥ 50 years old with BMI ≥ 30 kg/m^2 (n=69), female participants aged < 50 years old with BMI < 30 kg/m^2 (n=66), female participants aged < 50 years old with BMI ≥ 30 kg/m^2 (n=97), female participants aged ≥ 50 years old with BMI < 30 kg/m^2 (n=89), female participants aged ≥ 50 years old with BMI ≥ 30 kg/m^2 (n=76). Participant was flagged if greater than 20% of the metabolites for that participant were also flagged as outliers. Metabolite was flagged if greater than 5% of the participants' values for that metabolite were also flagged as outliers. Outliers were identified using Pearson residuals, DFFITS and Cooks D. Based on these measures and on the BA plots, no participants were flagged, whereas a total of 24 metabolites were flagged **(Table S1)**.

Within the replicated clusters, sphingomyelin (d18:1/24:1, d18:2/24:0)* from the sphingolipid metabolism & ceramides cluster as well as arginine and proline from the urea cycle-arginine-proline metabolism cluster were flagged by the BA method in PEAR **(Table S1)**. However, a further investigation)indicated that none of these three metabolites were flagged in PEAR-2.

Coefficient of Variation (CV)

CV for each metabolite was calculated by dividing standard deviation (SD) by the mean, to assess the variability of each metabolite across PEAR participants. The larger the metabolite's CV value, the higher is variability of that metabolite. The top 10% of the metabolites with the largest CV values (exceeding the CV cutoff of 0.778, n=36) were flagged **(Table S2)**.

Within the replicated clusters, 1-methylnicotinamide, pyridoxate and trigonelline from the cofactors and vitamins cluster were flagged by the CV QC step in PEAR **(Table S2)**. A further investigation showed that 1-methylnicotinamide and trigonelline had also large CV values in PEAR-2. However,

sensitivity analyses on these two metabolites (excluding the outlying participants) generated similar estimates and false discovery rates to that shown in **Table S5**.

References:

1. Johnson JA, Boerwinkle E, Zineh I, Chapman AB, Bailey K, Cooper-DeHoff RM, et al. Pharmacogenomics of antihypertensive drugs: rationale and design of the Pharmacogenomic Evaluation of Antihypertensive Responses (PEAR) study. Am Heart J. 2009;157(3):442-9.
2. Mehanna M, Gong Y, McDonough CW, Beitelshees AL, Gums JG, Chapman AB, et al. Blood pressure response to metoprolol and chlorthalidone in European and African Americans with hypertension. J Clin Hypertens (Greenwich). 2017;19(12):1301-8.
3. Pang Z, Chong J, Zhou G, de Lima Morais DA, Chang L, Barrette M, et al. MetaboAnalyst 5.0: narrowing the gap between raw spectra and functional insights. Nucleic Acids Res. 2021;49(W1):W388-W96.
4. Afgan E, Baker D, Batut B, van den Beek M, Bouvier D, Cech M, et al. The Galaxy platform for accessible, reproducible and collaborative biomedical analyses: 2018 update. Nucleic Acids Res. 2018;46(W1):W537-W44.
5. Bland JM, Altman DG. Statistical methods for assessing agreement between two methods of clinical measurement. Lancet. 1986;1(8476):307-10.
6. Kazmi N, Elliott HR, Burrows K, Tillin T, Hughes AD, Chaturvedi N, et al. Associations between high blood pressure and DNA methylation. PLoS One. 2020;15(1):e0227728.

Figure S1. Diagrams showing participants included in this study.

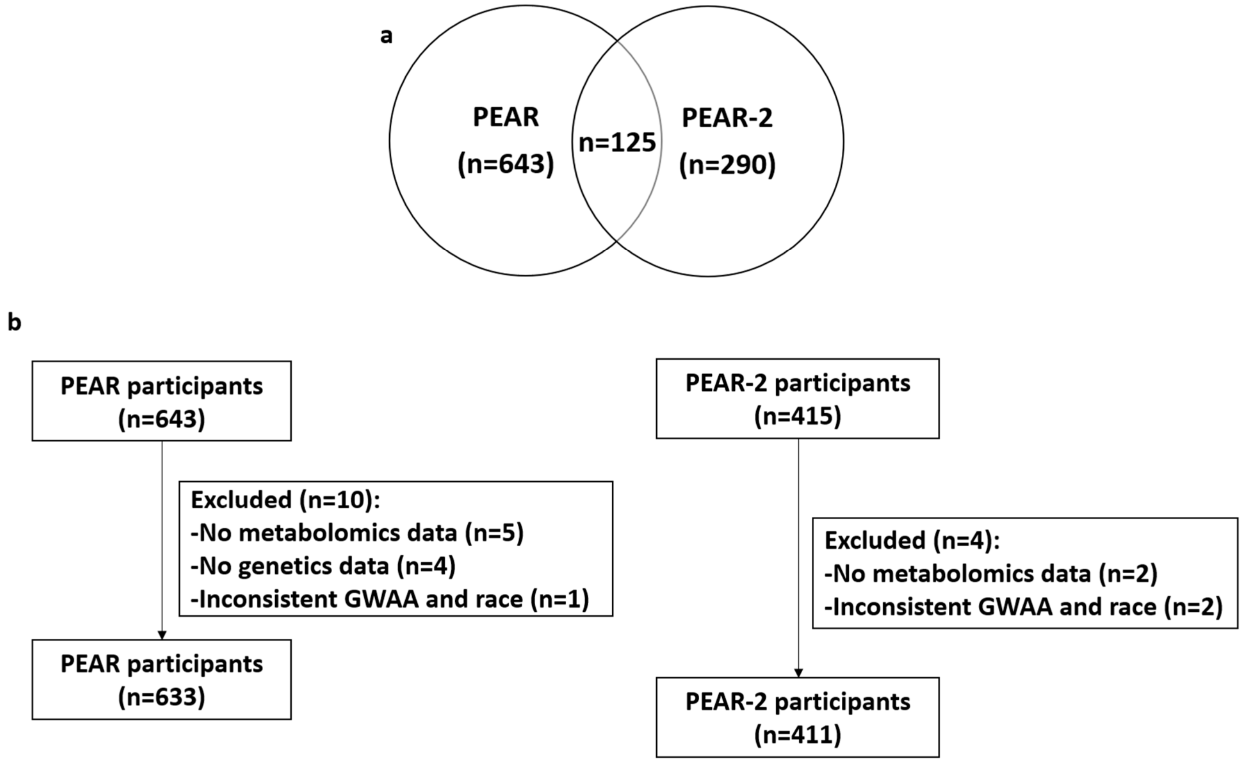

Figure S1a Venn diagram showing the overlapping participants who were enrolled in both PEAR and PEAR-2 trials (n=125). **Figure S1b** Consort diagrams showing PEAR and PEAR-2 participants included in this study. One participant from PEAR and two participants from PEAR-2 were excluded because they had inconsistent data for GWAA and race (identified by principal component analysis) (these participants were blacks with GWAA <45%). Abbreviations: PEAR, Pharmacogenomic Evaluation of Antihypertensive Responses; GWAA, Genetic West African ancestry.

Figure S2. Distributions of GWAA proportion among PEAR and PEAR-2 participants with GWAA < 45% and those with GWAA ≥ 45%

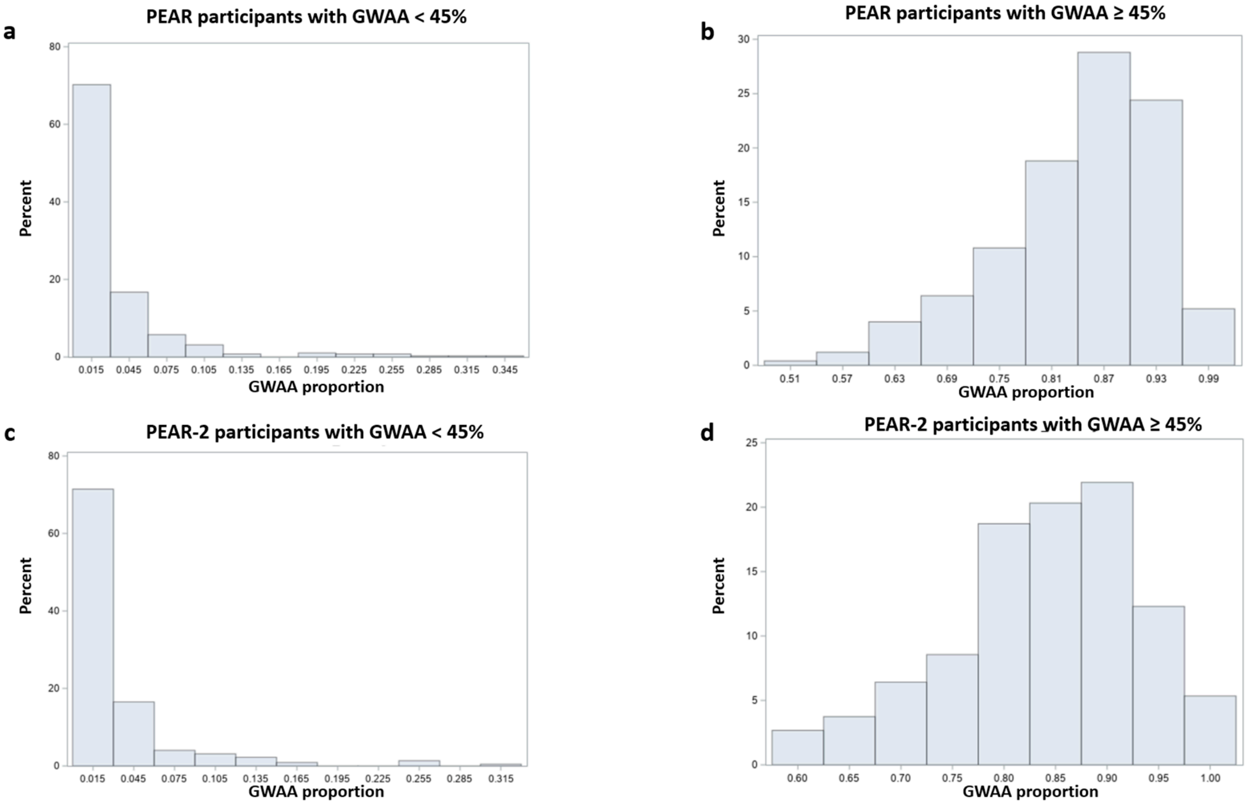

Figure S2a Histogram showing the distribution of GWAA proportion among PEAR participants with GWAA < 45% (n=383) (median 1.6%, IQR 1 – 3.4%; minimum 0.4%, maximum 34.6%). These participants included the following self-reported racial groups: White (n=371), Asian (n=7) and other (n=5). **Figure S2b** Histogram showing the distribution of GWAA proportion among PEAR participants with GWAA ≥ 45% (n=250) (median 85.4%, IQR 78.8 – 90.6%; minimum 48.4%, maximum 99.6%). These participants included the following self-reported racial groups: Black (n=242), White (n=1) and other (n=7). **Figure S2c** Histogram showing the distribution of GWAA proportion among PEAR-2 participants with GWAA < 45% (n=224) (median 1.5%, IQR 0.9 – 3.6%; minimum 0.4%, maximum 31.3%). These participants included the following self-reported racial groups: White (n=220), Asian (n=2) and other (n=2). **Figure S2d** Histogram showing the distribution of GWAA proportion among PEAR-2 participants with GWAA ≥ 45% (n=187) (median 84.8%, IQR 79.2 – 90.5%; minimum 59.3%, maximum 99.5%). These participants included the following self-reported racial groups: Black (n=185), and other (n=2).

Abbreviations: PEAR, Pharmacogenomic Evaluation of Antihypertensive Responses; GWAA, Genetic West African ancestry; IQR, interquartile range.

Figure S3. PCA scatter plots showing clustering of PEAR participants (n=633) based on the first three PCs

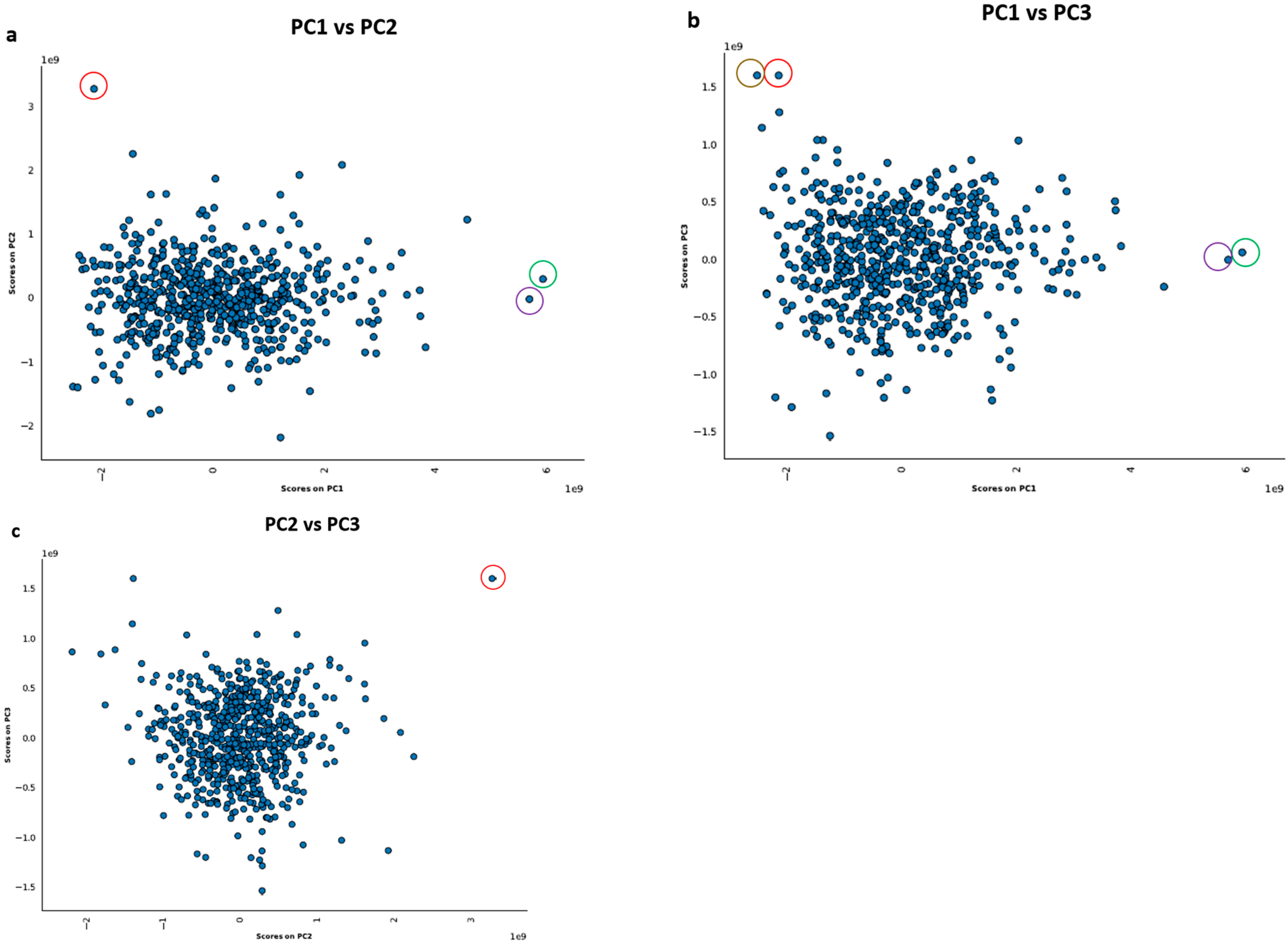

PCA scatter plots showing clustering of PEAR participants (n=633) based on a) PC1 vs. PC2, b) PC1 vs. PC3 and c) PC2 vs. PC3. PC1, PC2 and PC3 explain 50.5%, 11.2% and 6.1% of variability in the metabolomics data, respectively. Scatterplots show four outlying participants based on their metabolomics data (red, green, purple and brown circles). Abbreviations: PCA, principal component analysis; PEAR, Pharmacogenomic Evaluation of Antihypertensive Responses; PC, principal component.

Figure S4. Partial least-squares discriminant analysis (PLS-DA) of the 886 plasma metabolites in PEAR participants with GWAA < 45% (n=383) and PEAR participants with GWAA ≥ 45% (n=250)

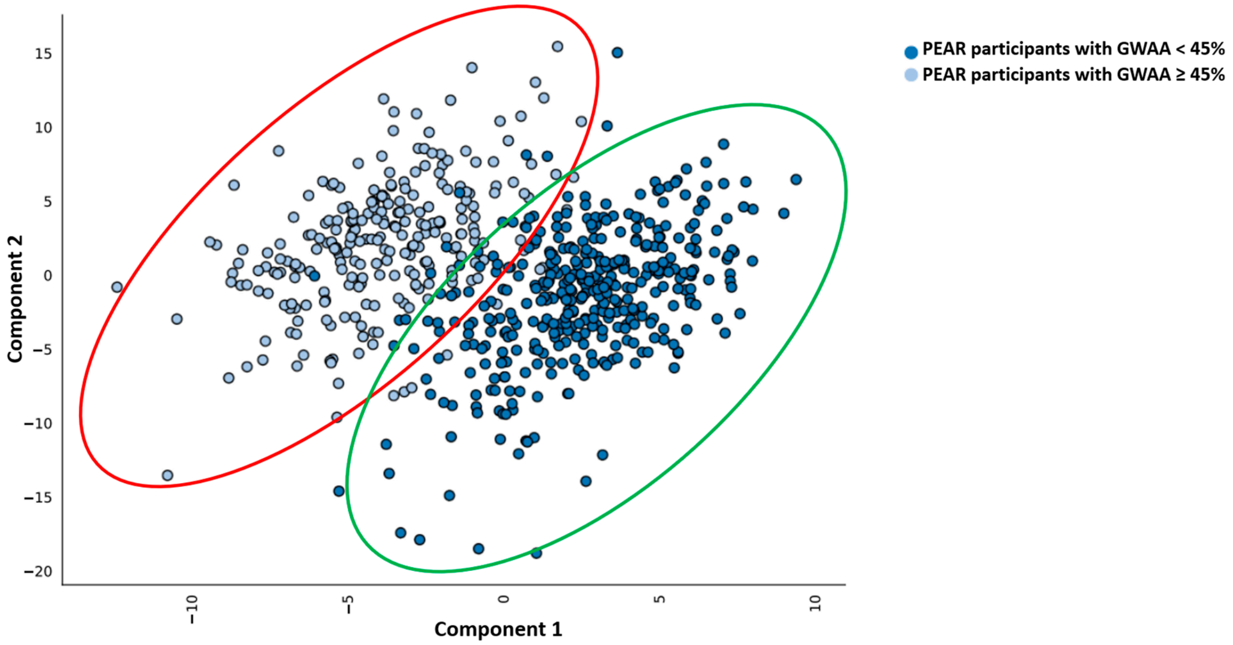

Two-dimensional score plot of PLS-DA model showing how the 886 plasma metabolites detected at baseline can discriminate the PEAR hypertensive participants with GWAA < 45% and PEAR hypertensive participants with GWAA ≥ 45%. Red circle indicates PEAR hypertensive participants with GWAA < 45%, while green circle indicates PEAR hypertensive participants with GWAA ≥ 45%.

Abbreviations: PEAR, Pharmacogenomic Evaluation of Antihypertensive Responses; GWAA, Genetic West African Ancestry.

Table S1. Metabolites flagged by the BA measures and plots in PEAR (n=24)

Metabolite	Classification[0]	Pathway
1-palmitoyl-2-arachidonoyl-GPC (16:0/20:4n6)	Lipid	Phosphatidylcholine (PC)
1-palmitoyl-2-linoleoyl-GPC (16:0/18:2)	Lipid	Phosphatidylcholine (PC)
1-palmitoyl-2-oleoyl-GPC (16:0/18:1)	Lipid	Phosphatidylcholine (PC)
1-palmitoyl-GPC (16:0)	Lipid	Lysophospholipid
1-stearoyl-2-linoleoyl-GPC (18:0/18:2)*	Lipid	Phosphatidylcholine (PC)
Arginine	Amino Acid	Urea cycle; Arginine and Proline Metabolism
Betaine	Amino Acid	Glycine, Serine and Threonine Metabolism
Carnitine	Lipid	Carnitine Metabolism
Creatine	Amino Acid	Creatine Metabolism
Creatinine	Amino Acid	Creatine Metabolism
Glucose	Carbohydrate	Glycolysis, Gluconeogenesis, and Pyruvate Metabolism
Glutamate	Amino Acid	Glutamate Metabolism
Glutamine	Amino Acid	Glutamate Metabolism
Isoleucine	Amnio Acid	Leucine, Isoleucine and Valine Metabolism
Leucine	Amnio Acid	Leucine, Isoleucine and Valine Metabolism
linoleate (18:2n6)	Lipid	Polyunsaturated Fatty Acid (n3 and n6)
Lysine	Amino Acid	Lysine Metabolism
oleate/vaccenate (18:1)	Lipid	Long Chain Fatty Acid
palmitate (16:0)	Lipid	Long Chain Fatty Acid
Phenylalanine	Amino Acid	Phenylalanine Metabolism
Proline	Amino Acid	Urea cycle; Arginine and Proline Metabolism
sphingomyelin (d18:1/24:1, d18:2/24:0)*	Lipid	Sphingolipid Metabolism
stearate (18:0)	Lipid	Long Chain Fatty Acid
X - 24803	NA	NA

* Indicates compounds that have not been officially confirmed based on a standard, but we are confident in its identity

[0] Metabolites were classified based on the Human Metabolome Database superclass classification http://www.hmdb.ca/classyfication

The underlined metabolites are those included within the replicated metabolic clusters.

Abbreviations: BA, Bland-Altman; NA, not applicable.

Table S2. The top 10% of metabolites with the largest CV values in PEAR (n=36)

Metabolite	Classification[0]	Pathway
16a-hydroxy DHEA 3-sulfate	Lipid	Steroid
1-methylnicotinamide	Cofactors and Vitamins	Nicotinate and Nicotinamide Metabolism
2-hydroxy-3-methylvalerate	Amino Acid	Leucine, Isoleucine and Valine Metabolism
3-carboxy-4-methyl-5-propyl-2-furanpropanoate (CMPF)	Lipid	Fatty Acid, Dicarboxylate
3-hydroxybutyrate (BHBA)	Lipid	Ketone Bodies
3-methylhistidine	Amino Acid	Histidine Metabolism
alpha-hydroxyisovalerate	Amino Acid	Leucine, Isoleucine and Valine Metabolism
adenosine 5'-monophosphate (AMP)	Nucleotide	Purine Metabolism, Adenine containing
androstenediol (3beta,17beta) disulfate (1)	Lipid	Steroid
arabitol/xylitol	Carbohydrate	Pentose Metabolism
bilirubin (E,E)*	Cofactors and Vitamins	Hemoglobin and Porphyrin Metabolism
erucate (22:1n9)	Lipid	Long Chain Fatty Acid
gamma-glutamylglycine	Peptide	Gamma-glutamyl Amino Acid
Glycochenodeoxycholate	Lipid	Primary Bile Acid Metabolism
hexanoylcarnitine (C6)	Lipid	Fatty Acid Metabolism (Acyl Carnitine)
Indoleacetate	Amino Acid	Tryptophan Metabolism
mannitol/sorbitol	Carbohydrate	Fructose, Mannose and Galactose Metabolism
Nicotinamide	Cofactors and Vitamins	Nicotinate and Nicotinamide Metabolism
N-methylproline	Amino Acid	Urea cycle; Arginine and Proline Metabolism
octanoylcarnitine (C8)	Lipid	Fatty Acid Metabolism (Acyl Carnitine)
oleoyl-linoleoyl-glycerol (18:1/18:2) [1]	Lipid	Diacylglycerol
phenol sulfate	Amino Acid	Tyrosine Metabolism
Pipecolate	Amino Acid	Lysine Metabolism
pregnen-diol disulfate*	Lipid	Steroid
Pyridoxate	Cofactors and Vitamins	Vitamin B6 Metabolism
trigonelline (N'-methylnicotinate)	Cofactors and Vitamins	Nicotinate and Nicotinamide Metabolism

trimethylamine N-oxide	Lipid	Phospholipid Metabolism
tryptophan betaine	Amino Acid	Tryptophan Metabolism
X - 09789, retired for 2,6-dihydroxybenzoic acid	Unknown	Unknown
X - 11440, retired for pregnenetriol disulfate*	Unknown	Unknown
X - 11441, retired for bilirubin degradation product, C17H20N2O5 (1)**	Unknown	Unknown
X - 11442, retired for bilirubin degradation product, C17H20N2O5 (2)**	Unknown	Unknown
X - 12101	NA	NA
X - 12462	NA	NA
X - 14838, retired for 1-methyl-5-imidazoleacetate	Unknown	Unknown
X - 24422	NA	NA

* Indicates compounds that have not been officially confirmed based on a standard, but we are confident in its identity

[0] Metabolites were classified based on the Human Metabolome Database superclass classification http://www.hmdb.ca/classyfication

The underlined metabolites are those included within the replicated metabolic clusters.

Abbreviations: CV, coefficient of variation; NA, not applicable.

Table S3. Classifications of the 423 differential metabolites (FDR < 0.2) between PEAR participants with GWAA <45% and those with GWAA ≥45% (screening phase)

Metabolite Classification/ Pathway	Metabolites with higher abundance in PEAR participants with GWAA ≥45% (n=209), N (%)	Metabolites with higher abundance in PEAR participants with GWAA <45% (n=214), N (%)
**Unknown**	50 (23.9%)	40 (18.7%)
**Unclassified known**	22 (10.5%)	18 (8.4%)
**Carbohydrate**	2 (1%)	3 (1.4%)
**Cofactors and Vitamins**	2 (1%)	9 (4.2%)
**Nucleotide**	5 (2.3%)	9 (4.2%)
**Peptide**	4 (1.9%)	4 (1.9%)
**Energy**	2 (1%)	0 (0%)
**Amino Acid**	41 (19.6%)	40 (18.7%)
-Alanine and Aspartate Metabolism	1 (2.4%)	3 (7.5%)
-Creatine Metabolism	3 (7.3%)	0 (0%)
-Glutamate Metabolism	2 (4.9%)	3 (7.5%)
-Glutathione Metabolism	0 (0%)	4 (10.0%)
-Glycine, Serine and Threonine Metabolism	4 (9.8%)	2 (5.0%)
-Guanidino and Acetamido Metabolism	1 (2.4%)	0 (0%)
-Histidine Metabolism	2 (4.9%)	2 (5.0%)
-Leucine, Isoleucine and Valine Metabolism	6 (14.6%)	4 (10.0%)
-Lysine Metabolism	2 (4.9%)	4 (10.0%)
-Methionine, Cysteine, SAM and Taurine Metabolism	2 (4.9%)	5 (12.5%)
-Phenylalanine Metabolism	1 (2.4%)	0 (0%)
-Polyamine Metabolism	0 (0%)	2 (5.0%)
-Tryptophan Metabolism	4 (9.8%)	5 (12.5%)
-Tyrosine Metabolism	6 (14.6%)	1 (2.5%)
-Urea cycle; Arginine and Proline Metabolism	7 (17.1%)	5 (12.5%)
**Lipid**	81 (38.8%)	91 (42.5%)
-Carnitine Metabolism	0 (0%)	1 (1.1%)
-Ceramide	2 (2.5%)	5 (5.5%)
-Diacylglycerol	2 (2.5%)	11 (12.1%)
-Endocannabinoid	2 (2.5%)	0 (0%)
-Fatty Acid Metabolism (Acyl Choline)	0 (0%)	1 (1.1%)
-Fatty Acid Metabolism (also BCAA Metabolism)	0 (0%)	2 (2.2%)
-Fatty Acid Metabolism (Acyl Glutamine)		
-Fatty Acid Metabolism (Acyl Carnitine)	1 (1.2%)	0 (0%)
-Fatty Acid Synthesis	2 (2.5%)	13 (14.3%)
-Fatty Acid	0 (0%)	1 (1.1%)
-Ketone Bodies	6 (7.4%)	5 (5.5%)
-Long Chain Fatty Acid	1 (1.2%)	0 (0%)
-Medium Chain Fatty Acid	5 (6.3%)	4 (4.4%)
-Lysophospholipid	0 (0%)	1 (1.1%)
-Lysoplasmalogen	6 (7.4%)	13 (14.3%)
-Monoacylglycerol	4 (4.9%)	0 (0%)
-Phosphatidylcholine (PC)	4 (4.9%)	1 (1.1%)
-Phosphatidylethanolamine (PE)	2 (2.5%)	8 (8.8%)
-Phosphatidylinositol (PI)	0 (0%)	4 (4.4%)

-Phospholipid Metabolism	0 (0%)	3 (3.3%)
-Plasmalogen	2 (2.5%)	0 (0%)
-Polyunsaturated Fatty Acid (n3 and n6)	10 (12.3%)	0 (0%)
-Bile Acid Metabolism	6 (7.4%)	0 (0%)
-Sphingolipid Metabolism	11 (13.6%)	2 (2.2%)
-Steroid	10 (12.3%)	12 (13.1%)
-Sterol	4 (4.9%)	4 (4.4%)
	1 (1.2%)	0 (0%)

Abbreviations: FDR, false discovery rate; PEAR, Pharmacogenomic Evaluation of Antihypertensive Responses.

Table S4. The 24 metabolic clusters created using the 353 PEAR metabolites identified from screening phase

Diacylglycerol & Monoacylglycerol (n=19)				
**Metabolite**	**Pathway**	**Estimate ± SE**	**FDR**	**Availability in PEAR-2**
palmitoleoyl-linoleoyl-glycerol (16:1/18:2) [1]*	Diacylglycerol	-0.54 ± 0.10	2.63E-06	Absent
linoleoyl-linoleoyl-glycerol (18:2/18:2) [1]*	Diacylglycerol	-0.33 ± 0.10	1.58E-03	Absent
linoleoyl-linolenoyl-glycerol (18:2/18:3) [2]*	Diacylglycerol	-0.32 ± 0.10	4.49E-03	Absent
oleoyl-oleoyl-glycerol (18:1/18:1) [1]*	Diacylglycerol	-0.32 ± 0.10	4.50E-03	Absent
oleoyl-linoleoyl-glycerol (18:1/18:2) [1]	Diacylglycerol	-0.28 ± 0.08	5.36E-03	Absent
oleoyl-oleoyl-glycerol (18:1/18:1) [2]*	Diacylglycerol	-0.33 ± 0.11	1.02E-02	Absent
palmitoyl-oleoyl-glycerol (16:0/18:1) [2]*	Diacylglycerol	-0.31 ± 0.10	1.52E-02	Absent
oleoyl-linoleoyl-glycerol (18:1/18:2) [2]	Diacylglycerol	-0.26 ± 0.09	2.00E-02	Absent
palmitoyl-linoleoyl-glycerol (16:0/18:2) [1]*	Diacylglycerol	-0.21 ± 0.10	8.23E-02	Absent
palmitoyl-oleoyl-glycerol (16:0/18:1) [1]*	Diacylglycerol	-0.23 ± 0.12	1.23E-01	Absent
palmitoyl-linoleoyl-glycerol (16:0/18:2) [2]*	Diacylglycerol	-0.18 ± 0.10	1.42E-01	Absent
linoleoyl-arachidonoyl-glycerol (18:2/20:4) [2]*	Diacylglycerol	0.15 ± 0.08	1.67E-01	Absent
linoleoyl-arachidonoyl-glycerol (18:2/20:4) [1]*	Diacylglycerol	0.14 ± 0.10	1.99E-01	Absent
1-arachidonylglycerol (20:4)	Monoacylglycerol	0.30 ± 0.06	6.04E-05	Present
2-linoleoylglycerol (18:2)	Monoacylglycerol	0.23 ± 0.10	7.30E-02	Present
1-palmitoleoylglycerol (16:1)*	Monoacylglycerol	-0.21 ± 0.10	1.20E-01	Present
1-linoleoylglycerol (18:2)	Monoacylglycerol	0.11 ± 0.06	1.44E-01	Present
1-dihomo-linolenylglycerol (20:3)	Monoacylglycerol	0.13 ± 0.07	1.84E-01	Present
X - 24035	unknown	-0.24 ± 0.10	6.64E-02	Absent
**Plasmalogen & Lysoplasmalogen (n=17)**				
**Metabolite**	**Pathway**	**Estimate ± SE**	**FDR**	**Availability in PEAR-2**
1-(1-enyl-palmitoyl)-2-arachidonoyl-GPE (P-16:0/20:4)*	Plasmalogen	0.37 ± 0.04	5.91E-14	Present

1-(1-enyl-palmitoyl)-2-arachidonoyl-GPE (P-16:0/20:4)*	Plasmalogen	0.32 ± 0.04	4.21E-11	Present
1-(1-enyl-palmitoyl)-2-arachidonoyl-GPC (P-16:0/20:4)*	Plasmalogen	0.23 ± 0.04	1.09E-08	Present
1-(1-enyl-palmitoyl)-2-palmitoyl-GPC (P-16:0/16:0)*	Plasmalogen	0.14 ± 0.03	7.67E-05	Present
1-(1-enyl-palmitoyl)-2-oleoyl-GPC (P-16:0/18:1)*	Plasmalogen	0.13 ± 0.03	1.21E-03	Present
1-(1-enyl-stearoyl)-2-oleoyl-GPE (P-18:0/18:1)	Plasmalogen	0.17 ± 0.05	1.87E-03	Present
1-(1-enyl-palmitoyl)-2-linoleoyl-GPC (P-16:0/18:2)*	Plasmalogen	0.12 ± 0.03	2.84E-03	Present
1-(1-enyl-palmitoyl)-2-oleoyl-GPE (P-16:0/18:1)*	Plasmalogen	0.13 ± 0.04	5.42E-03	Present
1-(1-enyl-palmitoyl)-2-linoleoyl-GPE (P-16:0/18:2)*	Plasmalogen	0.15 ± 0.05	8.60E-03	Present
1-(1-enyl-stearoyl)-2-linoleoyl-GPE (P-18:0/18:2)*	Plasmalogen	0.09 ± 0.05	1.35E-01	Present
1-(1-enyl-stearoyl)-GPE (P-18:0)*	Lysoplasmalogen	0.19 ± 0.05	3.37E-03	Present
1-(1-enyl-oleoyl)-GPE (P-18:1)*	Lysoplasmalogen	0.19 ± 0.06	9.32E-03	Present
1-(1-enyl-palmitoyl)-GPE (P-16:0)*	Lysoplasmalogen	0.16 ± 0.05	1.32E-02	Present
1-(1-enyl-palmitoyl)-GPC (P-16:0)*	Lysoplasmalogen	0.09 ± 0.04	1.19E-01	Present
X - 23587	unknown	0.58 ± 0.10	9.68E-07	Present
X - 24129	unknown	0.25 ± 0.06	7.56E-04	Absent
X - 23666	unknown	0.16 ± 0.05	6.16E-03	Absent
**PC, PE, PI, Lysophospholipid & Phospholipid Metabolism (n=48)**				
**Metabolite**	**Pathway**	**Estimate ± SE**	**FDR**	**Availability in PEAR-2**
1-palmitoyl-2-palmitoleoyl-GPC (16:0/16:1)*	Phosphatidylcholine (PC)	-0.36 ± 0.06	9.68E-07	Present
1-palmitoleoyl-2-linolenoyl-GPC (16:1/18:3)*	Phosphatidylcholine (PC)	-0.29 ± 0.06	3.60E-05	Absent
1-stearoyl-2-arachidonoyl-GPC (18:0/20:4)	Phosphatidylcholine (PC)	0.10 ± 0.02	2.20E-04	Present
1-palmitoyl-2-linoleoyl-GPC (16:0/18:2)	Phosphatidylcholine (PC)	-0.07 ± 0.02	4.27E-04	Present
1,2-dilinoleoyl-GPC (18:2/18:2)	Phosphatidylcholine (PC)	-0.14 ± 0.04	2.77E-03	Present
1-palmitoyl-2-oleoyl-GPC (16:0/18:1)	Phosphatidylcholine (PC)	-0.09 ± 0.03	1.04E-02	Present

1-stearoyl-2-linoleoyl-GPC (18:0/18:2)*	Phosphatidylcholine (PC)	-0.05 ± 0.02	2.32E-02	Present
1-palmitoyl-2-arachidonoyl-GPC (16:0/20:4n6)	Phosphatidylcholine (PC)	0.06 ± 0.02	3.01E-02	Absent
1-linoleoyl-2-linolenoyl-GPC (18:2/18:3)*	Phosphatidylcholine (PC)	-0.18 ± 0.07	3.51E-02	Present
1-stearoyl-2-oleoyl-GPC (18:0/18:1)	Phosphatidylcholine (PC)	-0.07 ± 0.04	1.38E-01	Present
1-stearoyl-2-linoleoyl-GPE (18:0/18:2)*	Phosphatidylethanolamine (PE)	-0.28 ± 0.06	2.11E-04	Present
1-palmitoyl-2-linoleoyl-GPE (16:0/18:2)	Phosphatidylethanolamine (PE)	-0.29 ± 0.07	2.94E-04	Present
1-oleoyl-2-linoleoyl-GPE (18:1/18:2)*	Phosphatidylethanolamine (PE)	-0.22 ± 0.07	5.83E-03	Absent
1-palmitoyl-2-oleoyl-GPE (16:0/18:1)	Phosphatidylethanolamine (PE)	-0.13 ± 0.07	1.69E-01	Present
1-palmitoyl-2-oleoyl-GPI (16:0/18:1)*	Phosphatidylinositol (PI)	-0.24 ± 0.07	5.36E-03	Present
1-palmitoyl-2-arachidonoyl-GPI (16:0/20:4)*	Phosphatidylinositol (PI)	-0.17 ± 0.06	3.00E-02	Present
1-palmitoyl-2-linoleoyl-GPI (16:0/18:2)	Phosphatidylinositol (PI)	-0.12 ± 0.05	7.11E-02	Present
1-palmitoleoyl-GPC (16:1)*	Lysophospholipid	-0.29 ± 0.05	3.00E-08	Present
1-linoleoyl-GPE (18:2)*	Lysophospholipid	-0.29 ± 0.05	4.85E-08	Present
2-palmitoleoyl-GPC (16:1)*	Lysophospholipid	-0.35 ± 0.08	1.81E-04	Present
1-linolenoyl-GPC (18:3)*	Lysophospholipid	-0.25 ± 0.06	4.63E-04	Present
1-palmitoyl-GPE (16:0)	Lysophospholipid	-0.18 ± 0.05	8.85E-04	Present
1-stearoyl-GPE (18:0)	Lysophospholipid	-0.16 ± 0.04	2.77E-03	Present
1-arachidonoyl-GPI (20:4)*	Lysophospholipid	0.15 ± 0.05	5.36E-03	Present
1-arachidonoyl-GPC (20:4n6)*	Lysophospholipid	0.12 ± 0.04	8.01E-03	Present
1-palmitoyl-GPC (16:0)	Lysophospholipid	-0.09 ± 0.03	8.07E-03	Present
1-linoleoyl-GPI (18:2)*	Lysophospholipid	0.16 ± 0.05	9.82E-03	Present
2-palmitoyl-GPC (16:0)*	Lysophospholipid	-0.17 ± 0.06	1.49E-02	Present
1-oleoyl-GPE (18:1)	Lysophospholipid	-0.14 ± 0.05	2.45E-02	Present
1-linoleoyl-GPC (18:2)	Lysophospholipid	-0.08 ± 0.03	3.96E-02	Present
1-lignoceroyl-GPC (24:0)	Lysophospholipid	-0.12 ± 0.05	4.93E-02	Present
1-oleoyl-GPC (18:1)	Lysophospholipid	-0.07 ± 0.03	4.98E-02	Present
2-stearoyl-GPE (18:0)*	Lysophospholipid	-0.14 ± 0.06	6.53E-02	Present
1-stearoyl-GPI (18:0)	Lysophospholipid	0.12 ± 0.06	8.28E-02	Present
1-linoleoyl-GPG (18:2)*	Lysophospholipid	0.10 ± 0.05	1.40E-01	Present
1-oleoyl-GPI (18:1)*	Lysophospholipid	0.13 ± 0.07	1.44E-01	Present

choline phosphate	Phospholipid Metabolism	0.07 ± 0.04	1.23E-01	Present
Glycerophosphoethanolamine	Phospholipid Metabolism	0.07 ± 0.04	1.94E-01	Present
X - 12729	unknown	0.87 ± 0.08	5.91E-14	Present
X - 12063, retired for metabolonic lactone sulfate	unknown	-0.58 ± 0.09	5.51E-08	Present
X - 13728	unknown	-0.64 ± 0.13	8.63E-06	Present
X - 19141	unknown	-0.27 ± 0.07	2.41E-03	Present
X - 16071, retired for 3-formylindole	unknown	-0.14 ± 0.05	1.91E-02	Present
X - 24435	unknown	0.13 ± 0.05	2.29E-02	Absent
X - 14314, retired for pyroglutamylleucine*	unknown	0.22 ± 0.08	3.27E-02	Present
X - 24542, retired for vanillic acid glycine	unknown	-0.48 ± 0.20	5.10E-02	Present
X - 21383	unknown	-0.17 ± 0.08	1.03E-01	Present
X - 09789, retired for 2,6-dihydroxybenzoic acid	unknown	-0.16 ± 0.09	1.69E-01	Present
**Sphingolipid Metabolism & Ceramides (n=33)**				
**Metabolite**	**Pathway**	**Estimate ± SE**	**FDR**	**Availability in PEAR-2**
sphingomyelin (d18:1/14:0, d16:1/16:0)*	Sphingolipid Metabolism	-0.18 ± 0.03	7.31E-09	Present
myristoyl dihydrosphingomyelin (d18:0/14:0)*	Sphingolipid Metabolism	-0.24 ± 0.04	3.99E-07	Absent
sphingomyelin (d18:2/14:0, d18:1/14:1)*	Sphingolipid Metabolism	-0.26 ± 0.05	1.46E-06	Present
sphingomyelin (d18:1/25:0, d19:0/24:1, d20:1/23:0, d19:1/24:0)*	Sphingolipid Metabolism	-0.25 ± 0.05	3.47E-05	Absent
sphingomyelin (d17:2/16:0, d18:2/15:0)*	Sphingolipid Metabolism	-0.22 ± 0.05	1.08E-04	Absent
sphingomyelin (d17:1/16:0, d18:1/15:0, d16:1/17:0)*	Sphingolipid Metabolism	-0.14 ± 0.03	1.83E-04	Absent
sphingomyelin (d18:2/24:1, d18:1/24:2)*	Sphingolipid Metabolism	0.10 ± 0.03	1.33E-03	Present
sphingomyelin (d18:1/18:1, d18:2/18:0)	Sphingolipid Metabolism	0.10 ± 0.03	2.39E-03	Present
sphingomyelin (d18:1/21:0, d17:1/22:0, d16:1/23:0)*	Sphingolipid Metabolism	-0.13 ± 0.04	1.28E-02	Present
N-nervonoyl-hexadecasphingosine (d16:1/24:1)*	Sphingolipid Metabolism	-0.35 ± 0.12	1.84E-02	Absent

sphingomyelin (d18:2/16:0, d18:1/16:1)*	Sphingolipid Metabolism	0.06 ± 0.02	3.25E-02	Present
sphingomyelin (d18:2/18:1)*	Sphingolipid Metabolism	0.12 ± 0.04	3.47E-02	Absent
sphingomyelin (d18:1/20:0, d16:1/22:0)*	Sphingolipid Metabolism	-0.07 ± 0.03	3.55E-02	Present
stearoyl sphingomyelin (d18:1/18:0)	Sphingolipid Metabolism	0.07 ± 0.03	4.01E-02	Present
sphingomyelin (d18:0/20:0, d16:0/22:0)*	Sphingolipid Metabolism	-0.15 ± 0.06	4.51E-02	Absent
sphingomyelin (d18:1/19:0, d19:1/18:0)*	Sphingolipid Metabolism	-0.11 ± 0.05	5.23E-02	Absent
N-palmitoyl-sphingadienine (d18:2/16:0)*	Sphingolipid Metabolism	-0.09 ± 0.04	5.32E-02	Absent
sphingomyelin (d18:1/24:1, d18:2/24:0)*	Sphingolipid Metabolism	0.07 ± 0.03	8.24E-02	Present
sphingomyelin (d18:2/24:2)*	Sphingolipid Metabolism	0.06 ± 0.03	1.16E-01	Absent
palmitoyl sphingomyelin (d18:1/16:0)	Sphingolipid Metabolism	0.04 ± 0.02	1.16E-01	Present
lignoceroyl sphingomyelin (d18:1/24:0)	Sphingolipid Metabolism	0.07 ± 0.04	1.42E-01	Absent
sphingomyelin (d18:1/20:1, d18:2/20:0)*	Sphingolipid Metabolism	0.06 ± 0.03	1.44E-01	Present
ceramide (d18:1/14:0, d16:1/16:0)*	Ceramides	-0.42 ± 0.05	2.22E-13	Absent
glycosyl ceramide (d18:1/20:0, d16:1/22:0)*	Ceramides	-0.14 ± 0.04	4.15E-03	Absent
ceramide (d18:2/24:1, d18:1/24:2)*	Ceramides	-0.12 ± 0.05	5.14E-02	Absent
ceramide (d18:1/20:0, d16:1/22:0, d20:1/18:0)*	Ceramides	-0.17 ± 0.07	5.22E-02	Absent
N-palmitoyl-sphingosine (d18:1/16:0)	Ceramides	-0.09 ± 0.04	6.34E-02	Present
glycosyl-N-(2-hydroxynervonoyl)-sphingosine (d18:1/24:1(2OH))*	Ceramides	0.14 ± 0.07	1.05E-01	Absent
glycosyl ceramide (d18:2/24:1, d18:1/24:2)*	Ceramides	0.09 ± 0.05	1.89E-01	Absent
X - 14939	unknown	0.35 ± 0.07	3.88E-06	Present
X - 18901	unknown	-0.53 ± 0.12	1.04E-04	Present
X - 24473	unknown	0.24 ± 0.12	1.09E-01	Absent
X - 24870	unknown	0.06 ± 0.03	1.52E-01	Absent
**Steroid & Sterol (n=12)**				

**Metabolite**	**Pathway**	**Estimate ± SE**	**FDR**	**Availability in PEAR-2**
androsterone sulfate	Steroid	-0.31 ± 0.09	5.93E-03	Present
5alpha-androstan-3alpha,17beta-diol monosulfate (1)	Steroid	-0.33 ± 0.10	8.01E-03	Present
5alpha-pregnan-3beta,20alpha-diol disulfate	Steroid	0.34 ± 0.13	2.96E-02	Present
pregnen-diol disulfate*	Steroid	0.19 ± 0.09	7.33E-02	Present
epiandrosterone sulfate	Steroid	-0.19 ± 0.09	9.49E-02	Present
pregnenolone sulfate	Steroid	0.18 ± 0.09	9.80E-02	Present
pregnanediol-3-glucuronide	Steroid	0.21 ± 0.11	1.25E-01	Present
5alpha-androstan-3beta,17alpha-diol disulfate	Steroid	-0.24 ± 0.14	1.83E-01	Present
Campesterol	Sterol	0.16 ± 0.07	8.56E-02	Absent
X - 24947	unknown	-0.66 ± 0.12	1.18E-06	Absent
X - 21258	unknown	-0.32 ± 0.12	3.65E-02	Present
X - 21668, retired for glyco-beta-muricholate**	unknown	0.34 ± 0.13	4.13E-02	Present
**Gamma-glutamyl Amino Acid (n=9)**				
**Metabolite**	**Pathway**	**Estimate ± SE**	**FDR**	**Availability in PEAR-2**
gamma-glutamylalanine	Peptide / Gamma-glutamyl Amino Acid	-0.22 ± 0.08	2.96E-02	Present
gamma-glutamylglutamine	Peptide / Gamma-glutamyl Amino Acid	-0.18 ± 0.07	4.08E-02	Present
gamma-glutamylthreonine	Peptide / Gamma-glutamyl Amino Acid	0.11 ± 0.05	7.11E-02	Absent
gamma-glutamyl-alpha-lysine	Peptide / Gamma-glutamyl Amino Acid	-0.10 ± 0.05	8.24E-02	Absent
gamma-glutamylvaline	Peptide / Gamma-glutamyl Amino Acid	0.12 ± 0.06	1.46E-01	Present
gamma-glutamylisoleucine*	Peptide / Gamma-glutamyl Amino Acid	0.11 ± 0.06	1.51E-01	Present
gamma-glutamyl-epsilon-lysine	Peptide / Gamma-glutamyl Amino Acid	-0.11 ± 0.06	1.58E-01	Present
HWESASXX*	Peptide / Polypeptide	0.25 ± 0.12	8.49E-02	Present
X - 24027	unknown	-0.56 ± 0.22	3.88E-02	Present
**Leucine, Isoleucine and Valine Metabolism (n=14)**				
**Metabolite**	**Pathway**	**Estimate ± SE**	**FDR**	**Availability in PEAR-2**

3-methylglutarylcarnitine (2)	Leucine, Isoleucine and Valine Metabolism	-0.74 ± 0.07	5.91E-14	Present
Ethylmalonate	Leucine, Isoleucine and Valine Metabolism	-0.31 ± 0.05	4.27E-07	Present
beta-hydroxyisovalerate	Leucine, Isoleucine and Valine Metabolism	0.28 ± 0.05	1.46E-06	Present
alpha-hydroxyisocaproate	Leucine, Isoleucine and Valine Metabolism	0.16 ± 0.04	4.22E-04	Present
3-hydroxy-2-ethylpropionate	Leucine, Isoleucine and Valine Metabolism	-0.16 ± 0.04	2.17E-03	Present
3-methylglutaconate	Leucine, Isoleucine and Valine Metabolism	-0.20 ± 0.05	2.56E-03	Present
alpha-hydroxyisovalerate	Leucine, Isoleucine and Valine Metabolism	0.22 ± 0.07	7.16E-03	Present
2-hydroxy-3-methylvalerate	Leucine, Isoleucine and Valine Metabolism	0.18 ± 0.06	1.20E-02	Present
N-acetylleucine	Leucine, Isoleucine and Valine Metabolism	0.16 ± 0.06	1.74E-02	Present
isovalerylcarnitine (C5)	Leucine, Isoleucine and Valine Metabolism	0.12 ± 0.05	4.06E-02	Absent
X - 13553	unknown	0.43 ± 0.05	1.33E-12	Present
X - 24845	unknown	0.34 ± 0.13	3.28E-02	Absent
X - 24293, retired for ethyl-alpha-glucopyranose	unknown	0.41 ± 0.20	1.04E-01	Present
X - 24422	unknown	0.14 ± 0.07	1.28E-01	Present
**Primary & Secondary Bile Acid Metabolism (n=24)**				
**Metabolite**	**Pathway**	**Estimate ± SE**	**FDR**	**Availability in PEAR-2**
glycochenodeoxycholate glucuronide (1)	Primary Bile Acid Metabolism	0.55 ± 0.13	2.10E-04	Present
glycochenodeoxycholate sulfate	Primary Bile Acid Metabolism	0.42 ± 0.13	7.11E-03	Present
Taurocholate	Primary Bile Acid Metabolism	0.45 ± 0.15	1.02E-02	Present
glycocholate	Primary Bile Acid Metabolism	0.26 ± 0.13	1.10E-01	Present
Taurochenodeoxycholate	Primary Bile Acid Metabolism	0.25 ± 0.13	1.24E-01	Present
taurocholenate sulfate	Secondary Bile Acid Metabolism	0.35 ± 0.09	7.45E-04	Present

glycodeoxycholate sulfate	Secondary Bile Acid Metabolism	0.44 ± 0.11	8.43E-04	Present
Isoursodeoxycholate	Secondary Bile Acid Metabolism	-0.56 ± 0.15	1.40E-03	Absent
glycocholenate sulfate*	Secondary Bile Acid Metabolism	0.26 ± 0.07	1.63E-03	Present
Ursodeoxycholate	Secondary Bile Acid Metabolism	-0.50 ± 0.16	1.02E-02	Present
taurolithocholate 3-sulfate	Secondary Bile Acid Metabolism	0.26 ± 0.12	9.19E-02	Present
Glycohyocholate	Secondary Bile Acid Metabolism	0.24 ± 0.13	1.44E-01	Present
glycolithocholate sulfate*	Secondary Bile Acid Metabolism	0.22 ± 0.12	1.65E-01	Present
X - 16935	unknown	0.54 ± 0.09	7.50E-08	Present
X - 18899	unknown	0.28 ± 0.07	2.94E-04	Present
X - 11880	unknown	0.21 ± 0.06	4.93E-03	Present
X - 21471	unknown	0.29 ± 0.09	4.98E-03	Present
X - 11378	unknown	0.20 ± 0.07	1.54E-02	Present
X - 24748	unknown	0.17 ± 0.06	2.78E-02	Present
X - 17162	unknown	0.47 ± 0.19	4.06E-02	Absent
X - 11308	unknown	0.13 ± 0.05	6.00E-02	Present
X - 11372	unknown	0.13 ± 0.06	6.62E-02	Present
X - 21339	unknown	0.13 ± 0.06	1.16E-01	Present
X - 12851	unknown	0.31 ± 0.17	1.61E-01	Present
**Cofactors and Vitamins (n=23)**				
**Metabolite**	**Pathway**	**Estimate ± SE**	**FDR**	**Availability in PEAR-2**
trigonelline (N'-methylnicotinate)	Nicotinate and Nicotinamide Metabolism	-0.75 ± 0.14	1.87E-06	Present
N1-Methyl-2-pyridone-5-carboxamide	Nicotinate and Nicotinamide Metabolism	-0.16 ± 0.06	2.64E-02	Present
1-methylnicotinamide	Nicotinate and Nicotinamide Metabolism	-0.11 ± 0.07	1.99E-01	Present
L-urobilin	Hemoglobin and Porphyrin Metabolism	0.78 ± 0.19	6.57E-04	Present
gamma-CEHC	Tocopherol Metabolism	-0.31 ± 0.08	1.57E-03	Present
alpha-tocopherol	Tocopherol Metabolism	-0.11 ± 0.04	4.33E-02	Present
gamma-CEHC glucuronide*	Tocopherol Metabolism	-0.24 ± 0.10	5.32E-02	Present
gamma-tocopherol/beta-tocopherol	Tocopherol Metabolism	0.12 ± 0.06	1.30E-01	Present

Pantothenate	Pantothenate and CoA Metabolism	-0.22 ± 0.06	1.09E-03	Present
retinol (Vitamin A)	Vitamin A Metabolism	-0.14 ± 0.04	3.54E-03	Present
Pyridoxate	Vitamin B6 Metabolism	-0.29 ± 0.10	1.61E-02	Present
X - 11485, retired for sulfate of piperine metabolite C18H21NO3 (1)*	Unknown	0.49 ± 0.11	1.01E-04	Present
X - 12511, retired for N-acetyl-2-aminooctanoate	Unknown	0.32 ± 0.07	2.87E-04	Present
X - 12544	Unknown	0.97 ± 0.24	7.32E-04	Present
X - 11452, retired for sulfate of piperine metabolite C16H19NO3 (2)*	Unknown	0.39 ± 0.11	2.40E-03	Present
X - 12231, retired for sulfate of piperine metabolite C16H19NO3 (3)*	Unknown	0.34 ± 0.11	8.20E-03	Present
X - 11852	Unknown	0.59 ± 0.19	1.12E-02	Present
X - 12013	Unknown	0.63 ± 0.24	3.47E-02	Present
X - 11850	Unknown	0.66 ± 0.27	4.89E-02	Present
X - 11843	Unknown	0.59 ± 0.26	6.50E-02	Present
X - 21849, retired for glycine conjugate of C10H14O2 (1)*	Unknown	0.18 ± 0.09	1.35E-01	Present
X - 17685	Unknown	-0.36 ± 0.19	1.42E-01	Present
X - 14662, retired for glycoursodeoxycholate sulfate (1)	Unknown	0.32 ± 0.18	1.65E-01	Present
**Pyrimidine & Purine Metabolism (n=14)**				
**Metabolite**	**Pathway**	**Estimate ± SE**	**FDR**	**Availability in PEAR-2**
N4-acetylcytidine	Pyrimidine Metabolism, Cytidine containing	-0.35 ± 0.06	2.04E-06	Present
Cytidine	Pyrimidine Metabolism, Cytidine containing	-0.20 ± 0.06	3.65E-03	Present
2'-O-methyluridine	Pyrimidine Metabolism, Uracil containing	0.21 ± 0.06	4.93E-03	Absent
N-acetyl-beta-alanine	Pyrimidine Metabolism, Uracil containing	-0.11 ± 0.04	1.73E-02	Present
Pseudouridine	Pyrimidine Metabolism, Uracil containing	-0.06 ± 0.03	9.92E-02	Present
Uridine	Pyrimidine Metabolism, Uracil containing	-0.07 ± 0.03	1.04E-01	Present

3-ureidopropionate	Pyrimidine Metabolism, Uracil containing	0.13 ± 0.07	1.28E-01	Present
5-methyluridine (ribothymidine)	Pyrimidine Metabolism, Uracil containing	-0.06 ± 0.03	1.91E-01	Present
3-aminoisobutyrate	Pyrimidine Metabolism, Thymine containing	-0.22 ± 0.07	6.16E-03	Present
Inosine	Purine Metabolism, (Hypo)Xanthine/Inosine containing	0.25 ± 0.09	1.55E-02	Present
Hypoxanthine	Purine Metabolism, (Hypo)Xanthine/Inosine containing	0.24 ± 0.09	2.71E-02	Present
N1-methylinosine	Purine Metabolism, (Hypo)Xanthine/Inosine containing	-0.09 ± 0.04	8.47E-02	Present
N1-methyladenosine	Purine Metabolism, Adenine containing	-0.05 ± 0.02	5.40E-02	Present
adenosine 5'-monophosphate (AMP)	Purine Metabolism, Adenine containing	0.16 ± 0.08	1.19E-01	Present
**Urea cycle; Arginine and Proline Metabolism (n=12)**				
**Metabolite**	**Pathway**	**Estimate ± SE**	**FDR**	**Availability in PEAR-2**
N-delta-acetylornithine	Urea cycle; Arginine and Proline Metabolism	-0.43 ± 0.07	1.29E-07	Present
N-acetylcitrulline	Urea cycle; Arginine and Proline Metabolism	0.65 ± 0.11	4.27E-07	Present
trans-4-hydroxyproline	Urea cycle; Arginine and Proline Metabolism	0.31 ± 0.05	4.27E-07	Present
Ornithine	Urea cycle; Arginine and Proline Metabolism	-0.16 ± 0.03	4.97E-07	Present
Homoarginine	Urea cycle; Arginine and Proline Metabolism	0.26 ± 0.05	9.68E-07	Present
N-acetylarginine	Urea cycle; Arginine and Proline Metabolism	0.28 ± 0.05	2.45E-06	Present
Proline	Urea cycle; Arginine and Proline Metabolism	-0.12 ± 0.03	1.15E-04	Present
Homocitrulline	Urea cycle; Arginine and Proline Metabolism	0.18 ± 0.07	2.68E-02	Present
N-acetylproline	Urea cycle; Arginine and Proline Metabolism	-0.20 ± 0.08	2.78E-02	Present
Arginine	Urea cycle; Arginine and Proline Metabolism	0.07 ± 0.03	3.00E-02	Present

dimethylarginine (SDMA + ADMA)	Urea cycle; Arginine and Proline Metabolism	-0.05 ± 0.02	5.29E-02	Present
N2,N5-diacetylornithine	Urea cycle; Arginine and Proline Metabolism	0.18 ± 0.09	1.16E-01	Present
**Tyrosine Metabolism (n=7)**				
**Metabolite**	**Pathway**	**Estimate ± SE**	**FDR**	**Availability in PEAR-2**
3-methoxytyrosine	Tyrosine Metabolism	0.18 ± 0.04	1.26E-05	Present
Vanillactate	Tyrosine Metabolism	0.28 ± 0.07	1.62E-03	Absent
3-(4-hydroxyphenyl)lactate	Tyrosine Metabolism	0.15 ± 0.04	4.44E-03	Present
gentisate	Tyrosine Metabolism	-0.31 ± 0.11	1.54E-02	Present
dopamine 3-O-sulfate	Tyrosine Metabolism	0.19 ± 0.07	2.98E-02	Absent
N-acetyltyrosine	Tyrosine Metabolism	0.12 ± 0.06	1.23E-01	Present
tyramine O-sulfate	Tyrosine Metabolism	0.26 ± 0.15	1.69E-01	Present
**Glutamate Metabolism (n=5)**				
**Metabolite**	**Pathway**	**Estimate ± SE**	**FDR**	**Availability in PEAR-2**
N-acetylglutamine	Gluatamate Metabolism	0.27 ± 0.07	7.32E-04	Present
beta-citrylglutamate	Gluatamate Metabolism	-0.16 ± 0.04	1.09E-03	Absent
Glutamine	Gluatamate Metabolism	-0.07 ± 0.02	8.07E-03	Present
N-acetylglutamate	Gluatamate Metabolism	0.11 ± 0.05	5.95E-02	Present
pyroglutamine*	Gluatamate Metabolism	-0.13 ± 0.06	1.16E-01	Present
**Glycine, Serine and Threonine Metabolism (n=9)**				
**Metabolite**	**Pathway**	**Estimate ± SE**	**FDR**	**Availability in PEAR-2**
Sarcosine	Glycine, Serine and Threonine Metabolism	-0.13 ± 0.04	6.16E-03	Present
Dimethylglycine	Glycine, Serine and Threonine Metabolism	0.12 ± 0.04	1.36E-02	Present
Threonine	Glycine, Serine and Threonine Metabolism	0.08 ± 0.03	3.26E-02	Present
Glycine	Glycine, Serine and Threonine Metabolism	-0.08 ± 0.03	5.39E-02	Present
N-acetylglycine	Glycine, Serine and Threonine Metabolism	0.13 ± 0.07	1.26E-01	Present
Betaine	Glycine, Serine and Threonine Metabolism	0.05 ± 0.03	1.64E-01	Present
X - 24806	unknown	-0.05 ± 0.02	1.93E-02	Absent
X - 24804	unknown	-0.05 ± 0.02	3.00E-02	Absent
X - 24803	unknown	-0.02 ± 0.01	5.75E-02	Absent
**Glutathione Metabolism (n=5)**				

Metabolite	Pathway	Estimate ± SE	FDR	Availability in PEAR-2
cysteine-glutathione disulfide	Glutathione Metabolism	-0.40 ± 0.09	1.11E-04	Absent
5-oxoproline	Glutathione Metabolism	-0.12 ± 0.03	2.33E-04	Present
N-acetylphenylalanine	Phenylalanine Metabolism	0.19 ± 0.06	8.34E-03	Present
Cysteinylglycine	Glutathione Metabolism	-0.14 ± 0.07	1.09E-01	Present
cys-gly, oxidized	Glutathione Metabolism	-0.16 ± 0.09	1.64E-01	Present
**Methionine, Cysteine, SAM and Taurine Metabolism (n=7)**				
**Metabolite**	**Pathway**	**Estimate ± SE**	**FDR**	**Availability in PEAR-2**
hypotaurine	Methionine, Cysteine, SAM and Taurine Metabolism	-0.29 ± 0.07	7.03E-04	Present
Methionine	Methionine, Cysteine, SAM and Taurine Metabolism	0.07 ± 0.02	9.62E-03	Present
N-acetylmethionine	Methionine, Cysteine, SAM and Taurine Metabolism	0.11 ± 0.04	5.40E-02	Present
methionine sulfone	Methionine, Cysteine, SAM and Taurine Metabolism	-0.11 ± 0.05	6.90E-02	Present
S-methylcysteine	Methionine, Cysteine, SAM and Taurine Metabolism	-0.10 ± 0.05	1.18E-01	Present
S-methylcysteine sulfoxide	Methionine, Cysteine, SAM and Taurine Metabolism	-0.13 ± 0.07	1.37E-01	Absent
Cysteine	Methionine, Cysteine, SAM and Taurine Metabolism	-0.06 ± 0.04	1.99E-01	Present
**Lysine Metabolism (n=8)**				
**Metabolite**	**Pathway**	**Estimate ± SE**	**FDR**	**Availability in PEAR-2**
N-trimethyl 5-aminovalerate	Lysine Metabolism	-0.23 ± 0.04	2.75E-06	Absent
N6-acetyllysine	Lysine Metabolism	-0.16 ± 0.04	7.86E-05	Present
5-hydroxylysine	Lysine Metabolism	-0.19 ± 0.04	1.00E-04	Present
Lysine	Lysine Metabolism	-0.08 ± 0.02	3.18E-04	Present
6-oxopiperidine-2-carboxylate	Lysine Metabolism	0.15 ± 0.06	2.82E-02	Absent
N-acetyl-cadaverine	Lysine Metabolism	0.36 ± 0.18	1.15E-01	Present
X - 13835, retired for 1-methyl-5-imidazolelactate	unknown	0.27 ± 0.10	2.69E-02	Present
X - 23652	unknown	0.19 ± 0.08	5.87E-02	Present
**Alanine and Aspartate Metabolism (n=4)**				
**Metabolite**	**Pathway**	**Estimate ± SE**	**FDR**	**Availability in PEAR-2**
alanine	Alanine and Aspartate Metabolism	-0.14 ± 0.03	3.98E-05	Present

Asparagine	Alanine and Aspartate Metabolism	-0.05 ± 0.02	7.15E-02	Present
N-acetylaspartate (NAA)	Alanine and Aspartate Metabolism	0.05 ± 0.03	1.69E-01	Present
Aspartate	Alanine and Aspartate Metabolism	-0.07 ± 0.04	1.99E-01	Present
**Histidine Metabolism (n=4)**				
**Metabolite**	**Pathway**	**Estimate ± SE**	**FDR**	**Availability in PEAR-2**
N-acetyl-1-methylhistidine*	Histidine Metabolism	0.44 ± 0.08	1.74E-06	Present
1-methylimidazoleacetate	Histidine Metabolism	-0.12 ± 0.03	3.51E-03	Present
hydantoin-5-propionic acid	Histidine Metabolism	-0.21 ± 0.08	2.37E-02	Present
1-methylhistidine	Histidine Metabolism	0.13 ± 0.05	2.68E-02	Present
**Carbohydrate (n=5)**				
**Metabolite**	**Pathway**	**Estimate ± SE**	**FDR**	**Availability in PEAR-2**
Mannose	Fructose, Mannose and Galactose Metabolism	0.09 ± 0.03	1.11E-02	Present
erythronate*	Aminosugar Metabolism	-0.08 ± 0.03	1.19E-02	Present
N-acetylglucosamine/N-acetylgalactosamine	Aminosugar Metabolism	0.08 ± 0.03	1.20E-02	Present
1,5-anhydroglucitol (1,5-AG)	Glycolysis, Gluconeogenesis, and Pyruvate Metabolism	-0.11 ± 0.04	3.28E-02	Present
arabonate/xylonate	Pentose Metabolism	-0.12 ± 0.05	4.06E-02	Present
**Creatine Metabolism (n=3)**				
**Metabolite**	**Pathway**	**Estimate ± SE**	**FDR**	**Availability in PEAR-2**
Creatinine	Creatine Metabolism	0.08 ± 0.02	5.56E-04	Present
Creatine	Creatine Metabolism	0.13 ± 0.05	2.34E-02	Present
guanidinoacetate	Creatine Metabolism	0.07 ± 0.04	1.35E-01	Present
**Fatty Acid (n=41)**				
**Metabolite**	**Pathway**	**Estimate ± SE**	**FDR**	**Availability in PEAR-2**
glutarate (pentanedioate)	Fatty Acid, Dicarboxylate	0.42 ± 0.08	9.70E-06	Present
Tetradecanedioate	Fatty Acid, Dicarboxylate	-0.25 ± 0.06	5.27E-04	Present
3-carboxy-4-methyl-5-propyl-2-furanpropanoate (CMPF)	Fatty Acid, Dicarboxylate	0.56 ± 0.15	1.49E-03	Present
Dodecanedioate	Fatty Acid, Dicarboxylate	-0.17 ± 0.05	2.92E-03	Present
Maleate	Fatty Acid, Dicarboxylate	-0.16 ± 0.09	1.45E-01	Present
linoleoyl ethanolamide	Endocannabinoid	0.25 ± 0.06	5.19E-04	Present
oleoyl ethanolamide	Endocannabinoid	0.11 ± 0.05	9.73E-02	Present

stearate (18:0)	Long Chain Fatty Acid	0.14 ± 0.04	1.05E-03	Present
myristoleate (14:1n5)	Long Chain Fatty Acid	-0.24 ± 0.07	3.37E-03	Present
pentadecanoate (15:0)	Long Chain Fatty Acid	-0.08 ± 0.03	3.25E-02	Present
arachidate (20:0)	Long Chain Fatty Acid	0.07 ± 0.03	7.51E-02	Present
eicosenoate (20:1)	Long Chain Fatty Acid	0.13 ± 0.07	1.15E-01	Present
myristate (14:0)	Long Chain Fatty Acid	-0.11 ± 0.05	1.16E-01	Present
margarate (17:0)	Long Chain Fatty Acid	0.09 ± 0.05	1.45E-01	Present
erucate (22:1n9)	Long Chain Fatty Acid	0.11 ± 0.06	1.60E-01	Present
palmitoleate (16:1n7)	Long Chain Fatty Acid	-0.14 ± 0.08	1.92E-01	Present
arachidonate (20:4n6)	Polyunsaturated Fatty Acid (n3 and n6)	0.18 ± 0.05	1.21E-03	Present
docosahexaenoate (DHA; 22:6n3)	Polyunsaturated Fatty Acid (n3 and n6)	0.24 ± 0.06	1.95E-03	Present
docosapentaenoate (n6 DPA; 22:5n6)	Polyunsaturated Fatty Acid (n3 and n6)	0.19 ± 0.06	6.29E-03	Present
docosadienoate (22:2n6)	Polyunsaturated Fatty Acid (n3 and n6)	0.14 ± 0.06	4.40E-02	Absent
dihomo-linoleate (20:2n6)	Polyunsaturated Fatty Acid (n3 and n6)	0.14 ± 0.06	6.98E-02	Present
adrenate (22:4n6)	Polyunsaturated Fatty Acid (n3 and n6)	0.13 ± 0.07	1.26E-01	Present
15-methylpalmitate	Fatty Acid, Branched	-0.13 ± 0.05	2.31E-02	Present
9-hydroxystearate	Fatty Acid, Monohydroxy	-0.22 ± 0.09	4.03E-02	Absent
3-hydroxydecanoate	Fatty Acid, Monohydroxy	0.14 ± 0.06	6.90E-02	Present
alpha-hydroxycaproate	Fatty Acid, Monohydroxy	0.17 ± 0.08	8.15E-02	Absent
3-hydroxylaurate	Fatty Acid, Monohydroxy	0.13 ± 0.06	8.49E-02	Present
laurate (12:0)	Medium Chain Fatty Acid	-0.17 ± 0.05	6.22E-03	Present
2-aminoheptanoate	Fatty Acid, Amino	0.15 ± 0.05	1.27E-02	Present
acetoacetate	Ketone Bodies	0.29 ± 0.13	8.53E-02	Absent
X - 02269, retired for hydroxy-CMPF*	unknown	0.49 ± 0.14	2.56E-03	Present
X - 21353	unknown	0.22 ± 0.06	3.64E-03	Present
X - 21607	unknown	0.23 ± 0.07	7.83E-03	Present
X - 23293, retired for cis-4-decenoate	unknown	0.25 ± 0.08	8.38E-03	Present
X - 13866	unknown	0.23 ± 0.09	3.73E-02	Present
X - 11438, retired for tridecenedioate (C13:1-DC)*	unknown	-0.20 ± 0.08	3.81E-02	Present
X - 12849	unknown	0.35 ± 0.15	6.51E-02	Present

X - 22764, retired for branched-chain, straight-chain, or cyclopropyl 12:1 fatty acid*	unknown	0.16 ± 0.07	8.01E-02	Present
X - 13891, retired for dodecenedioate (C12:1-DC)	unknown	0.24 ± 0.12	1.06E-01	Present
X - 17327, retired for glutamine conjugate of C7H12O2*	unknown	-0.24 ± 0.12	1.25E-01	Present
X - 12442, retired for tetradecadienoate (14:2)*	unknown	0.10 ± 0.06	1.99E-01	Present
**Fatty Acid Metabolism (Acyl Carnitine) (n=21)**				
**Metabolite**	**Pathway**	**Estimate ± SE**	**FDR**	**Availability in PEAR-2**
adipoylcarnitine (C6-DC)	Fatty Acid Metabolism (Acyl Carnitine)	-0.45 ± 0.07	1.09E-08	Absent
nervonoylcarnitine (C24:1)*	Fatty Acid Metabolism (Acyl Carnitine)	-0.20 ± 0.05	2.04E-04	Absent
butyrylcarnitine (C4)	Fatty Acid Metabolism (also BCAA Metabolism)	-0.20 ± 0.05	1.66E-03	Absent
behenoylcarnitine (C22)*	Fatty Acid Metabolism (Acyl Carnitine)	-0.22 ± 0.06	2.40E-03	Absent
margaroylcarnitine*	Fatty Acid Metabolism (Acyl Carnitine)	-0.17 ± 0.05	3.21E-03	Absent
lignoceroylcarnitine (C24)*	Fatty Acid Metabolism (Acyl Carnitine)	-0.17 ± 0.05	4.05E-03	Absent
dihomo-linolenoylcarnitine (20:3n3 or 6)*	Fatty Acid Metabolism (Acyl Carnitine)	-0.17 ± 0.05	5.52E-03	Absent
malonylcarnitine	Fatty Acid Synthesis	-0.15 ± 0.05	6.92E-03	Present
pimeloylcarnitine/3-methyladipoylcarnitine (C7-DC)	Fatty Acid Metabolism (Acyl Carnitine)	-0.21 ± 0.07	1.67E-02	Absent
hexanoylglutamine	Fatty Acid Metabolism (Acyl Glutamine)	0.29 ± 0.10	1.93E-02	Absent
myristoylcarnitine (C14)	Fatty Acid Metabolism (Acyl Carnitine)	-0.14 ± 0.05	3.01E-02	Absent
eicosenoylcarnitine (C20:1)*	Fatty Acid Metabolism (Acyl Carnitine)	-0.12 ± 0.05	4.35E-02	Absent
carnitine	Carnitine Metabolism	-0.04 ± 0.02	7.11E-02	Present
methylmalonate (MMA)	Fatty Acid Metabolism (also BCAA Metabolism)	-0.12 ± 0.06	9.19E-02	Present
palmitoleoylcarnitine (C16:1)*	Fatty Acid Metabolism (Acyl Carnitine)	-0.11 ± 0.05	1.07E-01	Absent

docosahexaenoylcarnitine (C22:6)*	Fatty Acid Metabolism (Acyl Carnitine)	0.16 ± 0.08	1.29E-01	Absent
oleoylcarnitine (C18:1)	Fatty Acid Metabolism (Acyl Carnitine)	-0.07 ± 0.04	1.61E-01	Absent
palmitoylcarnitine (C16)	Fatty Acid Metabolism (Acyl Carnitine)	-0.06 ± 0.04	1.69E-01	Absent
dihomo-linolenoyl-choline	Fatty Acid Metabolism (Acyl Choline)	-0.19 ± 0.11	1.69E-01	Present
ximenoylcarnitine (C26:1)*	Fatty Acid Metabolism (Acyl Carnitine)	-0.08 ± 0.05	1.70E-01	Absent
cis-4-decenoylcarnitine (C10:1)	Fatty Acid Metabolism (Acyl Carnitine)	0.11 ± 0.06	1.75E-01	Absent
**Tryptophan Metabolism (n=9)**				
**Metabolite**	**Pathway**	**Estimate ± SE**	**FDR**	**Availability in PEAR-2**
Kynurenine	Tryptophan Metabolism	-0.15 ± 0.03	5.72E-05	Present
tryptophan betaine	Tryptophan Metabolism	-0.65 ± 0.14	7.48E-05	Present
N-acetylkynurenine (2)	Tryptophan Metabolism	0.59 ± 0.14	3.20E-04	Present
N-formylanthranilic acid	Tryptophan Metabolism	0.37 ± 0.09	4.63E-04	Absent
5-bromotryptophan	Tryptophan Metabolism	-0.18 ± 0.05	1.65E-03	Present
Serotonin	Tryptophan Metabolism	0.36 ± 0.14	3.27E-02	Present
Indolepropionate	Tryptophan Metabolism	-0.27 ± 0.11	5.25E-02	Present
Indoleacetate	Tryptophan Metabolism	-0.17 ± 0.07	7.04E-02	Present
Indoleacetylglutamine	Tryptophan Metabolism	0.19 ± 0.11	1.85E-01	Present

A positive value in the estimates indicate a higher level of that metabolite in PEAR participants with GWAA ≥ 45% than those with GWAA < 45%. A negative value in the estimates indicate a higher level of that metabolite in PEAR participants with GWAA < 45% than those with GWAA ≥ 45%. Unknown metabolites within each metabolic cluster had moderate or strong correlations with one or more of the known classified metabolites within the same cluster based on MMC approach.

Abbreviations: PEAR, Pharmacogenomic Evaluation of Antihypertensive Responses; SE, standard error; FDR, false discovery rate; GWAA, Genetic West African ancestry; MMC, modulated modularity clustering.

Table S5. Metabolites within each of the 4 replicated metabolic clusters

Plasmalogen & Lysoplasmalogen (n=15)					
		PEAR		PEAR-2	
**Metabolite**	**Pathway**	**Estimate ± SE**	**FDR**	**Estimate ± SE**	**FDR**
1-(1-enyl-stearoyl)-2-arachidonoyl-GPE (P-18:0/20:4)*	Plasmalogen	0.37 ± 0.04	5.91E-14	0.31 ± 0.05	2.92E-07
1-(1-enyl-palmitoyl)-2-arachidonoyl-GPE (P-16:0/20:4)*	Plasmalogen	0.32 ± 0.04	4.21E-11	0.34 ± 0.06	8.14E-08
1-(1-enyl-palmitoyl)-2-arachidonoyl-GPC (P-16:0/20:4)*	Plasmalogen	0.23 ± 0.04	1.09E-08	0.23 ± 0.04	4.77E-07
1-(1-enyl-palmitoyl)-2-palmitoyl-GPC (P-16:0/16:0)*	Plasmalogen	0.14 ± 0.03	7.67E-05	0.04 ± 0.04	3.05E-01
1-(1-enyl-palmitoyl)-2-oleoyl-GPC (P-16:0/18:1)*	Plasmalogen	0.13 ± 0.03	1.21E-03	0.01 ± 0.04	8.74E-01
1-(1-enyl-stearoyl)-2-oleoyl-GPE (P-18:0/18:1)	Plasmalogen	0.17 ± 0.05	1.87E-03	0.13 ± 0.06	5.07E-02
1-(1-enyl-palmitoyl)-2-linoleoyl-GPC (P-16:0/18:2)*	Plasmalogen	0.12 ± 0.03	2.84E-03	0.05 ± 0.05	2.83E-01
1-(1-enyl-palmitoyl)-2-oleoyl-GPE (P-16:0/18:1)*	Plasmalogen	0.13 ± 0.04	5.42E-03	0.12 ± 0.05	4.92E-02
1-(1-enyl-palmitoyl)-2-linoleoyl-GPE (P-16:0/18:2)*	Plasmalogen	0.15 ± 0.05	8.60E-03	0.12 ± 0.06	8.86E-02
1-(1-enyl-stearoyl)-2-linoleoyl-GPE (P-18:0/18:2)*	Plasmalogen	0.09 ± 0.05	1.35E-01	0.04 ± 0.06	5.06E-01
1-(1-enyl-stearoyl)-GPE (P-18:0)*	Lysoplasmalogen	0.19 ± 0.05	3.37E-03	0.11 ± 0.07	1.30E-01
1-(1-enyl-oleoyl)-GPE (P-18:1)*	Lysoplasmalogen	0.19 ± 0.06	9.32E-03	0.10 ± 0.07	1.93E-01
1-(1-enyl-palmitoyl)-GPE (P-16:0)*	Lysoplasmalogen	0.16 ± 0.05	1.32E-02	0.14 ± 0.06	4.75E-02
1-(1-enyl-palmitoyl)-GPC (P-16:0)*	Lysoplasmalogen	0.09 ± 0.04	1.19E-01	-0.05 ± 0.05	3.64E-01
X - 23587	unknown	0.58 ± 0.10	9.68E-07	0.73 ± 0.16	4.16E-05
**Sphingolipid Metabolism & Ceramides (n=14)**					

		PEAR		PEAR-2	
**Metabolite**	**Pathway**	**Estimate ± SE**	**FDR**	**Estimate ± SE**	**FDR**
sphingomyelin (d18:1/14:0, d16:1/16:0)*	Sphingolipid Metabolism	-0.18 ± 0.03	7.31E-09	-0.24 ± 0.04	8.14E-08
sphingomyelin (d18:2/14:0, d18:1/14:1)*	Sphingolipid Metabolism	-0.26 ± 0.05	1.46E-06	-0.32 ± 0.05	1.84E-09
sphingomyelin (d18:2/24:1, d18:1/24:2)*	Sphingolipid Metabolism	0.10 ± 0.03	1.33E-03	0.10 ± 0.03	7.91E-03
sphingomyelin (d18:1/18:1, d18:2/18:0)	Sphingolipid Metabolism	0.10 ± 0.03	2.39E-03	0.12 ± 0.04	4.15E-03
sphingomyelin (d18:1/21:0, d17:1/22:0, d16:1/23:0)*	Sphingolipid Metabolism	-0.13 ± 0.04	1.28E-02	-0.13 ± 0.05	1.07E-02
sphingomyelin (d18:2/16:0, d18:1/16:1)*	Sphingolipid Metabolism	0.06 ± 0.02	3.25E-02	-0.01 ± 0.03	8.47E-01
sphingomyelin (d18:1/20:0, d16:1/22:0)*	Sphingolipid Metabolism	-0.07 ± 0.03	3.55E-02	-0.07 ± 0.03	6.68E-02
stearoyl sphingomyelin (d18:1/18:0)	Sphingolipid Metabolism	0.07 ± 0.03	4.01E-02	0.10 ± 0.03	9.12E-03
sphingomyelin (d18:1/24:1, d18:2/24:0)*	Sphingolipid Metabolism	0.07 ± 0.03	8.24E-02	0.05 ± 0.03	1.30E-01
palmitoyl sphingomyelin (d18:1/16:0)	Sphingolipid Metabolism	0.04 ± 0.02	1.16E-01	0.07 ± 0.03	7.91E-03
sphingomyelin (d18:1/20:1, d18:2/20:0)*	Sphingolipid Metabolism	0.06 ± 0.03	1.44E-01	0.09 ± 0.03	1.89E-02
N-palmitoyl-sphingosine (d18:1/16:0)	Ceramides	-0.09 ± 0.04	6.34E-02	-0.12 ± 0.04	9.27E-03
X – 14939	Unknown	0.35 ± 0.07	3.88E-06	0.29 ± 0.08	8.54E-04
X – 18901	Unknown	-0.53 ± 0.12	1.04E-04	-0.35 ± 0.15	3.45E-02
**Cofactors and Vitamins (n=22)**					
		**PEAR**		**PEAR-2**	
**Metabolite**	**Pathway**	**Estimate ± SE**	**FDR**	**Estimate ± SE**	**FDR**
gamma-CEHC	Tocopherol Metabolism	-0.31 ± 0.08	1.57E-03	-0.41 ± 0.10	8.00E-05
alpha-tocopherol	Tocopherol Metabolism	-0.11 ± 0.04	4.33E-02	0.12 ± 0.04	1.37E-02
gamma-CEHC glucuronide*	Tocopherol Metabolism	-0.24 ± 0.10	5.32E-02	-0.14 ± 0.12	2.78E-01
gamma-tocopherol/beta-tocopherol	Tocopherol Metabolism	0.12 ± 0.06	1.30E-01	0.16 ± 0.08	4.92E-02

trigonelline (N'-methylnicotinate)	Nicotinate and Nicotinamide Metabolism	-0.75 ± 0.14	1.87E-06	-0.77 ± 0.20	5.43E-04
N1-Methyl-2-pyridone-5-carboxamide	Nicotinate and Nicotinamide Metabolism	-0.16 ± 0.06	2.64E-02	-0.23 ± 0.08	9.33E-03
1-methylnicotinamide	Nicotinate and Nicotinamide Metabolism	-0.11 ± 0.07	1.99E-01	-0.28 ± 0.10	1.42E-02
pantothenate	Pantothenate and CoA Metabolism	-0.22 ± 0.06	1.09E-03	-0.30 ± 0.07	4.16E-05
retinol (Vitamin A)	Vitamin A Metabolism	-0.14 ± 0.04	3.54E-03	-0.16 ± 0.05	1.58E-03
pyridoxate	Vitamin B6 Metabolism	-0.29 ± 0.10	1.61E-02	-0.50 ± 0.13	3.23E-04
X - 11485, retired for sulfate of piperine metabolite C18H21NO3 (1)*	unknown	0.49 ± 0.11	1.01E-04	0.60 ± 0.15	2.38E-04
X - 12511, retired for N-acetyl-2-aminooctanoate	unknown	0.32 ± 0.07	2.87E-04	0.15 ± 0.09	1.16E-01
X - 12544	unknown	0.97 ± 0.24	7.32E-04	1.35 ± 0.33	1.99E-04
X - 11452, retired for sulfate of piperine metabolite C16H19NO3 (2)*	unknown	0.39 ± 0.11	2.40E-03	0.45 ± 0.16	8.44E-03
X - 12231, retired for sulfate of piperine metabolite C16H19NO3 (3)*	unknown	0.34 ± 0.11	8.20E-03	0.48 ± 0.15	5.17E-03
X – 11852	unknown	0.59 ± 0.19	1.12E-02	-0.31 ± 0.30	3.55E-01
X – 12013	unknown	0.63 ± 0.24	3.47E-02	0.91 ± 0.31	7.91E-03
X – 11850	unknown	0.66 ± 0.27	4.89E-02	1.12 ± 0.34	3.45E-03
X – 11843	unknown	0.59 ± 0.26	6.50E-02	0.84 ± 0.33	1.89E-02
X - 21849, retired for glycine conjugate of C10H14O2 (1)*	unknown	0.18 ± 0.09	1.35E-01	0.05 ± 0.10	6.87E-01
X – 17685	unknown	-0.36 ± 0.19	1.42E-01	-0.46 ± 0.26	1.07E-01
X - 14662, retired for glycoursodeoxycholate sulfate (1)	unknown	0.32 ± 0.18	1.65E-01	-0.26 ± 0.23	3.05E-01
**Urea cycle; Arginine and Proline Metabolism (n=12)**					
		**PEAR**		**PEAR-2**	
**Metabolite**	**Pathway**	**Estimate ± SE**	**FDR**	**Estimate ± SE**	**FDR**

N-delta-acetylornithine	Urea cycle; Arginine and Proline Metabolism	-0.43 ± 0.07	1.29E-07	-0.60 ± 0.09	1.32E-08
N-acetylcitrulline	Urea cycle; Arginine and Proline Metabolism	0.65 ± 0.11	4.27E-07	0.79 ± 0.15	3.68E-06
trans-4-hydroxyproline	Urea cycle; Arginine and Proline Metabolism	0.31 ± 0.05	4.27E-07	0.24 ± 0.06	7.83E-04
ornithine	Urea cycle; Arginine and Proline Metabolism	-0.16 ± 0.03	4.97E-07	-0.18 ± 0.03	1.32E-08
homoarginine	Urea cycle; Arginine and Proline Metabolism	0.26 ± 0.05	9.68E-07	0.18 ± 0.05	3.06E-03
N-acetylarginine	Urea cycle; Arginine and Proline Metabolism	0.28 ± 0.05	2.45E-06	0.31 ± 0.07	3.71E-05
proline	Urea cycle; Arginine and Proline Metabolism	-0.12 ± 0.03	1.15E-04	-0.12 ± 0.03	4.08E-04
homocitrulline	Urea cycle; Arginine and Proline Metabolism	0.18 ± 0.07	2.68E-02	0.004 ± 0.10	9.68E-01
N-acetylproline	Urea cycle; Arginine and Proline Metabolism	-0.20 ± 0.08	2.78E-02	-0.24 ± 0.08	8.44E-03
arginine	Urea cycle; Arginine and Proline Metabolism	0.07 ± 0.03	3.00E-02	0.08 ± 0.03	9.49E-03
dimethylarginine (SDMA + ADMA)	Urea cycle; Arginine and	-0.05 ± 0.02	5.29E-02	-0.10 ± 0.02	7.67E-05

	Proline Metabolism				
N2,N5-diacetylornithine	Urea cycle; Arginine and Proline Metabolism	0.18 ± 0.09	1.16E-01	0.01 ± 0.10	8.96E-01

A positive value in the estimates indicate a higher level of that metabolite in PEAR participants with GWAA ≥ 45% than those with GWAA < 45%. A negative value in the estimates indicate a higher level of that metabolite in PEAR participants with GWAA < 45% than those with GWAA ≥ 45%. Unknown metabolites within each metabolic cluster had moderate or strong correlations with one or more of the known classified metabolites within the same cluster based on MMC approach. The underlined metabolites are those which did not have the same direction of effect in PEAR-2 as in PEAR.

Abbreviations: PEAR, Pharmacogenomic Evaluation of Antihypertensive Responses; SE, standard error; FDR, false discovery rate; GWAA, Genetic West African ancestry; MMC, modulated modularity clustering.

Table S6. Metabolites within replicated clusters having same direction of effect using proportion of GWAA among PEAR participants with GWAA ≥45% (n=250) and PEAR-2 participants with GWAA ≥45% (n=187)

**Plasmalogen & Lysoplasmalogen (n=11)**						
			**PEAR participants with GWAA ≥ 45% (n=250)**		**PEAR-2 participants with GWAA ≥ 45% (n=187)**	
**Metabolite**	**Pathway**	**Direction of effect (Discovery & Replication)**	**Estimate ± SE**	**FDR**	**Estimate ± SE**	**FDR**
1-(1-enyl-stearoyl)-2-arachidonoyl-GPE (P-18:0/20:4)*	Plasmalogen	Higher in participants with GWAA ≥ 45%	0.53 ± 0.24	0.34	0.63 ± 0.28	0.72
1-(1-enyl-palmitoyl)-2-arachidonoyl-GPE (P-16:0/20:4)*	Plasmalogen	Higher in participants with GWAA ≥ 45%	0.44 ± 0.25	0.49	0.52 ± 0.30	0.72
1-(1-enyl-palmitoyl)-2-arachidonoyl-GPC (P-16:0/20:4)*	Plasmalogen	Higher in participants with GWAA ≥ 45%	0.19 ± 0.19	0.70	0.30 ± 0.19	0.72
1-(1-enyl-stearoyl)-2-oleoyl-GPE (P-18:0/18:1)	Plasmalogen	Higher in participants with GWAA ≥ 45%	0.26 ± 0.26	0.70	0.10 ± 0.31	0.92
1-(1-enyl-palmitoyl)-2-oleoyl-GPE (P-16:0/18:1)*	Plasmalogen	Higher in participants with GWAA ≥ 45%	0.39 ± 0.22	0.49	0.05 ± 0.28	0.95
1-(1-enyl-palmitoyl)-2-linoleoyl-GPE (P-16:0/18:2)*	Plasmalogen	Higher in participants with GWAA ≥ 45%	0.24 ± 0.25	0.70	0.18 ± 0.33	0.92
1-(1-enyl-stearoyl)-2-linoleoyl-GPE (P-18:0/18:2)*	Plasmalogen	Higher in participants with GWAA ≥ 45%	0.28 ± 0.25	0.70	0.16 ± 0.30	0.92
1-(1-enyl-stearoyl)-GPE (P-18:0)*	Lysoplasmalogen	Higher in participants	0.52 ± 0.30	0.49	0.17 ± 0.33	0.92

		with GWAA ≥ 45%				
1-(1-enyl-oleoyl)-GPE (P-18:1)*	Lysoplasmalogen	Higher in participants with GWAA ≥ 45%	0.48 ± 0.34	0.69	0.08 ± 0.35	0.93
1-(1-enyl-palmitoyl)-GPE (P-16:0)*	Lysoplasmalogen	Higher in participants with GWAA ≥ 45%	0.51 ± 0.31	0.50	0.19 ± 0.33	0.92
X - 23587	Unknown	Higher in participants with GWAA ≥ 45%	0.63 ± 0.52	0.70	0.81 ± 0.86	0.83
**Sphingolipid Metabolism & Ceramides (n=5)**						
			**PEAR participants with AA ≥ 45% (n=250)**		**PEAR-2 participants with AA ≥ 45% (n=187)**	
**Metabolite**	**Pathway**	**Direction of effect (Discovery & Replication)**	**Estimate ± SE**	**FDR**	**Estimate ± SE**	**FDR**
sphingomyelin (d18:1/20:1, d18:2/20:0)*	Sphingolipid Metabolism	Higher in participants with GWAA ≥ 45%	0.15 ± 0.19	0.75	0.09 ± 0.19	0.92
sphingomyelin (d18:1/14:0, d16:1/16:0)*	Sphingolipid Metabolism	Higher in participants with GWAA < 45%	-0.17 ± 0.15	0.70	-0.26 ± 0.22	0.81
sphingomyelin (d18:2/14:0, d18:1/14:1)*	Sphingolipid Metabolism	Higher in participants with GWAA < 45%	-0.29 ± 0.27	0.70	-0.37 ± 0.26	0.80
N-palmitoyl-sphingosine (d18:1/16:0)	Ceramides	Higher in participants with GWAA < 45%	-0.08 ± 0.19	0.89	-0.47 ± 0.21	0.72
X - 14939	Unknown	Higher in participants	0.64 ± 0.36	0.49	0.73 ± 0.40	0.72

		with GWAA ≥ 45%				
**Cofactors and Vitamins (n=8)**						
			**PEAR participants with AA ≥ 45% (n=250)**		**PEAR-2 participants with AA ≥ 45% (n=187)**	
**Metabolite**	**Pathway**	**Direction of effect (Discovery & Replication)**	**Estimate ± SE**	**FDR**	**Estimate ± SE**	**FDR**
gamma-tocopherol/beta-tocopherol	Tocopherol Metabolism	Higher in participants with GWAA ≥ 45%	0.24 ± 0.31	0.76	0.16 ± 0.39	0.92
alpha-tocopherol	Tocopherol Metabolism	Higher in participants with GWAA < 45%	-0.01 ± 0.30	0.99	-0.04 ± 0.20	0.93
trigonelline (N'-methylnicotinate)	Nicotinate and Nicotinamide Metabolism	Higher in participants with GWAA < 45%	-0.13 ± 0.74	0.94	-0.33 ± 0.97	0.92
pantothenate	Pantothenate and CoA Metabolism	Higher in participants with GWAA < 45%	-0.27 ± 0.33	0.75	-0.24 ± 0.32	0.87
pyridoxate	Vitamin B6 Metabolism	Higher in participants with GWAA < 45%	-0.24 ± 0.57	0.89	-0.17 ± 0.62	0.93
X - 12511, retired for N-acetyl-2-aminooctanoate	Unknown	Higher in participants with GWAA ≥ 45%	0.72 ± 0.42	0.49	0.29 ± 0.44	0.92
X - 21849, retired for glycine conjugate of C10H14O2 (1)*	Unknown	Higher in participants with GWAA ≥ 45%	1.03 ± 0.48	0.36	0.11 ± 0.53	0.93
X - 11843	Unknown	Higher in participants	0.81 ± 1.51	0.86	3.56 ± 1.99	0.72

		with GWAA ≥ 45%				
**Urea cycle; Arginine and Proline Metabolism (n=6)**						
			**PEAR participants with AA ≥ 45% (n=250)**		**PEAR-2 participants with AA ≥ 45% (n=187)**	
**Metabolite**	**Pathway**	**Direction of effect (Discovery & Replication)**	**Estimate ± SE**	**FDR**	**Estimate ± SE**	**FDR**
arginine	Urea cycle; Arginine and Proline Metabolism	Higher in participants with GWAA ≥ 45%	0.07 ± 0.14	0.89	0.11 ± 0.13	0.83
homoarginine	Urea cycle; Arginine and Proline Metabolism	Higher in participants with GWAA ≥ 45%	0.57 ± 0.26	0.34	0.35 ± 0.31	0.81
N-acetylcitrulline	Urea cycle; Arginine and Proline Metabolism	Higher in participants with GWAA ≥ 45%	1.73 ± 0.63	0.18	0.28 ± 0.87	0.92
N-acetylarginine	Urea cycle; Arginine and Proline Metabolism	Higher in participants with GWAA ≥ 45%	0.93 ± 0.32	0.18	0.15 ± 0.39	0.92
trans-4-hydroxyproline	Urea cycle; Arginine and Proline Metabolism	Higher in participants with GWAA ≥ 45%	0.76 ± 0.33	0.34	0.41 ± 0.37	0.81
proline	Urea cycle; Arginine and Proline Metabolism	Higher in participants with GWAA < 45%	-0.12 ± 0.14	0.75	-0.09 ± 0.15	0.92

A positive value in the estimates indicate that as proportion of GWAA increases among PEAR and PEAR-2 participants with GWAA ≥ 45%, the metabolite level increases, whereas a negative value in the estimates indicate that as proportion of GWAA increases among PEAR and PEAR-2 participants with GWAA ≥ 45%, the metabolite level decreases. Abbreviations: PEAR, Pharmacogenomic Evaluation of Antihypertensive Responses; SE, standard error; FDR, false discovery rate; GWAA, Genetic West African ancestry.

Figure S5. The KEGG nicotinate and nicotinamide metabolic pathway

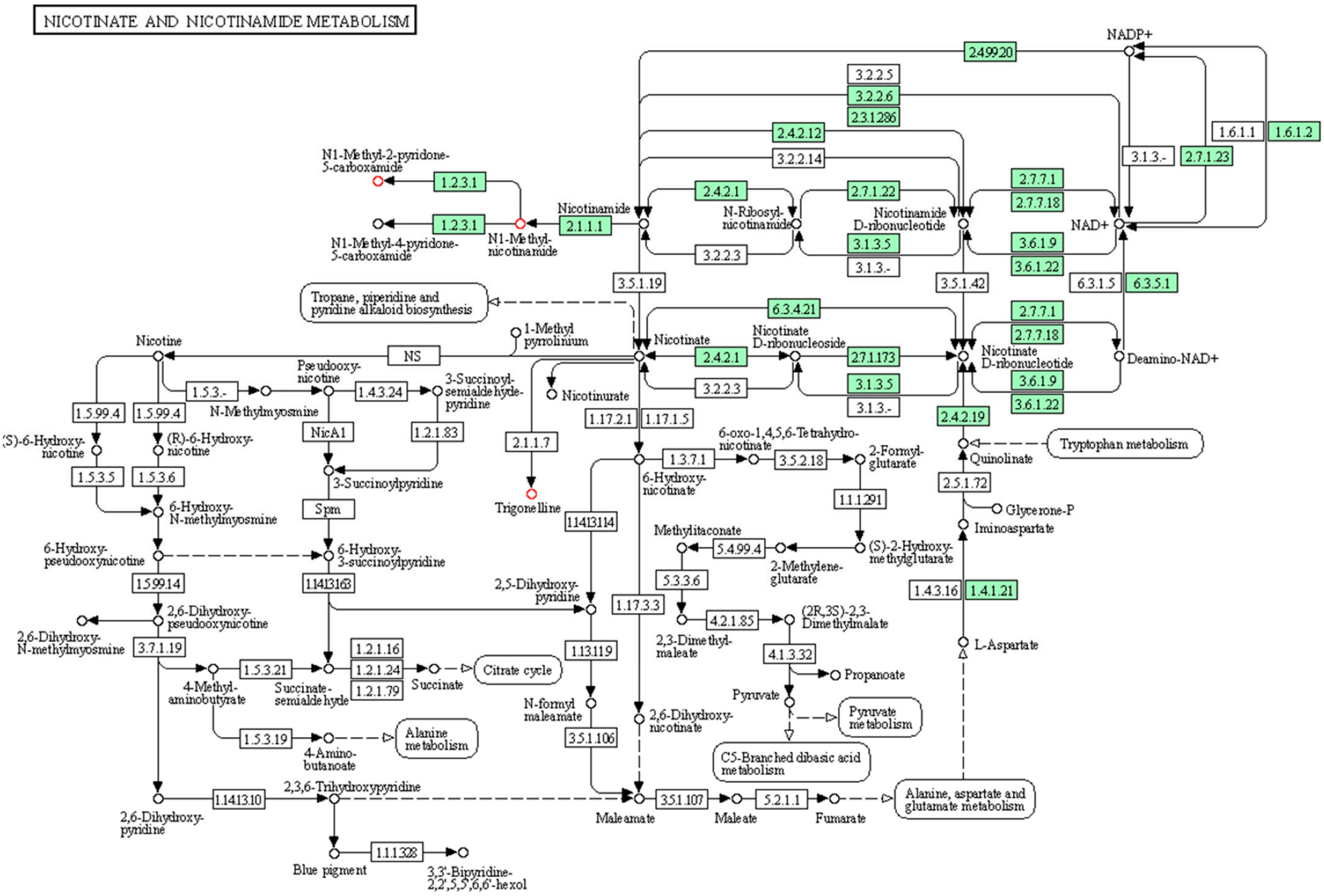

This chart represents the KEGG homo sapiens nicotinate and nicotinamide metabolism map (map00760). The metabolites circled with red are those that were included within the successfully replicated metabolic cluster (cofactors and vitamins): N1-Methyl-2-pyridone-5-carboxamide (KEGG ID C05842), 1-methylnicotinamide (KEGG ID C02918) and trigonelline (KEGG ID C01004). The 3 metabolites had higher abundance in PEAR and PEAR-2 participants with GWAA < 45%, compared to those with GWAA ≥ 45%.

Abbreviations: KEGG, Kyoto Encyclopedia of Genes and Genomes; PEAR, Pharmacogenomic Evaluation of Antihypertensive Responses; GWAA, Genetic West African ancestry.

Figure S6. The KEGG arginine and proline metabolic pathway

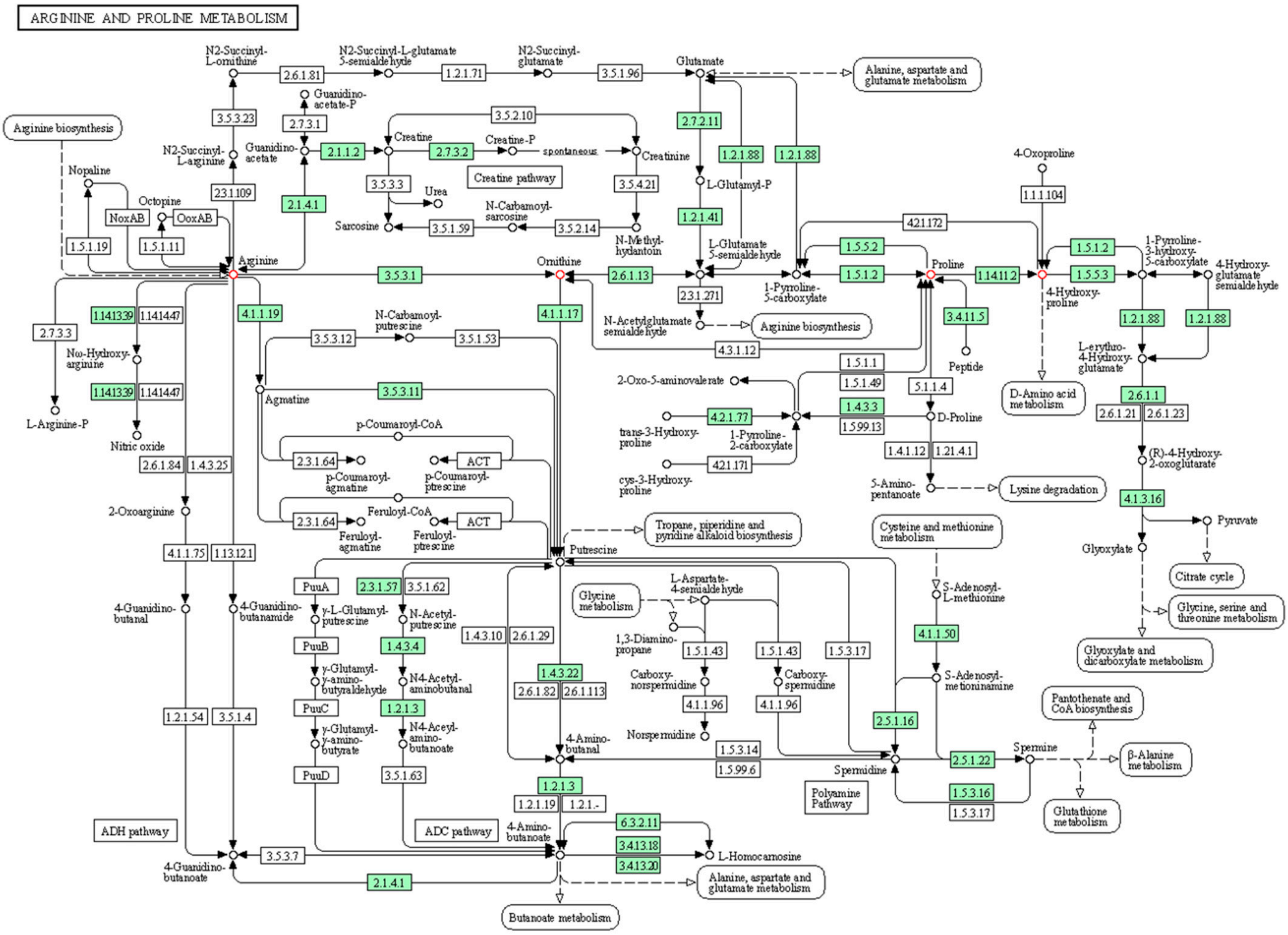

This chart represents the KEGG homo sapiens arginine and proline metabolism map (map00330). The metabolites circled with red are those that were included within the successfully replicated metabolic cluster (urea cycle; arginine and proline): arginine (KEGG ID C00062) and 4-hydroxyproline (KEGG ID C01157) had higher abundance in PEAR and PEAR-2 participants with GWAA ≥ 45% than those with GWAA < 45%, whereas ornithine (KEGG ID C00077) and proline (KEGG ID C00148) had higher abundance in PEAR and PEAR-2 participants with GWAA < 45% than those with GWAA ≥ 45%. The upstream gene to hydroxyproline (prolyl 4-hydroxylase subunit alpha 3 or P4HA3) (1.14.11.2) has been previously associated with systolic and diastolic blood pressure in South Asians [6].

Abbreviations: KEGG, Kyoto Encyclopedia of Genes and Genomes; PEAR, Pharmacogenomic Evaluation of Antihypertensive Responses; GWAA, Genetic West African ancestry.

Table S7. The 84 AIMs used to estimate proportion of GWAA for each PEAR and PEAR-2 participant

AIM	Chromosome	bp	EUR_AFR
rs2986742	1	6550376	0.67
rs647325	1	18170886	0.41
rs4908343	1	27931698	0.78
rs7554936	1	151122489	0.65
rs630101	1	236772416	0.85
rs6003	1	197031021	0.62
rs798443	2	7968275	0.80
rs7421394	2	14756349	0.63
rs4666200	2	29538411	0.64
rs13400937	2	79864923	0.70
rs260690	2	109579738	0.55
rs2339475	2	29999790	0.78
rs935925	2	237214545	0.77
rs9809104	3	39146429	0.73
rs6548616	3	79399575	0.71
rs9845457	3	135914476	0.60
rs4596126	3	13659897	0.73
rs1039524	3	114012274	0.44
rs10007810	4	41554364	0.71
rs385194	4	85309078	0.71
rs7657799	4	105375423	0.81
rs1525760	4	117135380	0.83
rs758973	4	13539373	0.73
rs37369	5	35037115	0.58
rs6451722	5	43711378	0.66
rs6556352	5	155471714	0.71
rs930072	5	36666071	0.85
rs185493	5	177991258	0.58
rs3317	5	112212151	0.49
rs1040045	6	4747159	0.63
rs2397060	6	51611470	0.68
rs192655	6	90518278	0.36
rs3024354	6	6292430	0.82
rs3734693	6	43965165	0.70
rs362949	6	146652354	0.67
rs793979	6	164766576	0.60
rs1029122	6	132250141	0.56
rs455726	6	111699368	0.50
rs7803075	7	130742066	0.71

rs6464211	7	151873853	0.72
rs3176921	8	67091379	0.47
rs285	8	19815189	0.43
rs1408801	9	12672320	0.48
rs913258	9	4877246	0.86
rs2301550	9	20354796	0.81
rs7349	10	31817905	0.95
rs1837606	11	15838137	0.42
rs11227699	11	66898492	0.48
rs948028	11	120644447	0.68
rs1042602	11	88911696	0.37
rs2416791	12	11701488	0.86
rs772262	12	56163734	0.81
rs2070586	12	109277720	0.30
rs1503767	12	118889488	0.64
rs9530435	13	75993887	0.72
rs9522149	13	111827167	0.73
rs895898	13	39227547	0.63
rs1760921	14	20818131	0.76
rs8021730	14	67886781	0.65
rs200354	14	99375321	0.51
rs3784230	14	105679055	0.58
rs179562	14	31224458	0.67
rs8035124	15	92105708	0.62
rs1374092	15	72808428	0.83
rs1800404	15	28235773	0.79
rs4984913	16	740466	0.68
rs2269793	16	19272908	0.50
rs7201030	16	76523792	0.80
rs2033111	17	53788280	0.43
rs11652805	17	62987151	0.84
rs8082034	17	53866575	0.70
rs2816	17	7923564	0.43
rs2891	17	3705526	0.42
rs7238445	18	49781544	0.73
rs4891825	18	67867663	0.80
rs874299	18	75056284	0.67
rs8113143	18	33652247	0.68
rs4436849	18	30555446	0.73
rs3745099	19	52901905	0.83
rs2532060	19	55614923	0.60

rs2835370	21	37885625	0.42
rs2064056	21	36595108	0.76
rs4821004	22	32366359	0.54
rs4821667	22	37830081	0.83

The bp was based on build 37 (GRCh37). EUR_AFR is the difference in allele frequency between the European and African populations.

Abbreviations: AIM, ancestry informative marker; GWAA, Genetic West African ancestry; PEAR, Pharmacogenomic Evaluation of Antihypertensive Responses; bp, base pair position; EUR, European; AFR, African.
